# Supplementary material for: Characterization of a thaumarchaeal symbiont that drives incomplete nitrification in the tropical sponge Ianthella basta
Source: Environ Microbiol. 2019 Jul 25;21(10):3831–54. doi: 10.1111/1462-2920.14732 (PMC6790972; doi:10.1111/1462-2920.14732)
Supplement: Supplementary file 1 — Appendix. Supporting Information. [file EMI-21-3831-s001.docx]

**Supporting Information**

**Characterization of a thaumarchaeal symbiont that drives incomplete nitrification in the tropical sponge *Ianthella basta***

Florian U. Moeller, Nicole S. Webster, Craig W. Herbold, Faris Behnam, Daryl Domman, Mads Albertsen, Maria Mooshammer^1^, Stephanie Markert, Dmitrij Turaev, Dörte Becher, Thomas Rattei, Thomas Schweder, Andreas Richter, Margarete Watzka, Per Halkjaer Nielsen, and Michael Wagner

**Table of contents:**

1. Supporting Results and Discussion

2. Supporting Experimental Procedures

3. Figures S1 – S9

4. Table S1-S5

5. References

**Supporting Results and Discussion**

**High GC content of *Ca.* Nitrosospongia ianthellae.** Both thaumarchaeal symbiont MAGs from *I. basta* possess the highest GC content (64.8%) of any genome-sequenced thaumarchaeote (Fig. 2C). While the GC content in the genome of *Ca.* Cenarchaeum symbiosum is similarly high (57.4%), the thaumarchaeal MAGs from the sponges *Cymbastela concentrica* (Moitinho-Silva *et al*., 2017a) and a glass sponge (Tian *et al*., 2016) had a much lower GC content (Fig. 2B). The high GC content found in the *I. basta* thaumarchaeal symbiont is consistent with the high GC content evident in Mediterranean sponge metagenomes (GC content 58-63%), particularly when compared to seawater metagenomes (GC content 41%) collected at the same location (Horn *et al*., 2016). Similarly, the GC content of six sponge microbiome metagenomes (Fan *et al*., 2012) that we queried had an average GC content of 57.8% ± 6.7(SD), with three great barrier reef sponge species having an average microbiome metagenomic GC content of 63.3% ± 2.2 (SD). In contrast, the average GC composition of selected marine and coral microbiomes are ~48% and ~45%, respectively (Reichenberger *et al*., 2015). While several environmental factors are thought to affect the genomic GC content of bacteria and archaea (Foerstner *et al*., 2005; Wang *et al*., 2006), increased rates of homologous recombination via GC-biased gene conversion has recently been proposed as a crucial factor universally influencing the nucleotide content of microbial genes and genomes (Lassalle *et al*., 2015). The possibility that sponge microbiomes are hot spots for input of novel genetic material via lateral gene transfer events (Fan *et al*., 2012; Horn *et al*., 2016), and also display increased homologous recombination rates should be assessed in future work.

**Environmental distribution of *Ca.* Nitrosospongia ianthellae**. The *Ca*. N. ianthellae 16S rRNA gene was queried against the Sponge Microbiome Project (SMP) database containing amplicon data sets from 268 sponge species including *I. basta* (Moitinho-Silva *et al*., 2017b). The top hits were inserted into our reference 16S rRNA gene tree (Fig. 2A) using the Evolutionary Placement Algorithm (EPA; Berger *et al*., 2011). 76 OTUs placed adjacent to the sponge-specific sequence cluster 174, including one with 100% identity to *Ca.* N. ianthellae. These 76 OTUs comprised, on average, 17.2% ± 7.1 (SD) of all reads obtained from *I. basta* individuals in the SMP dataset, consistent with abundances determined by FISH and qPCR (see main text). Interestingly, the *Ca*. N. ianthellae-adjacent OTUs were also found to comprise 0.25 – 7.5% of the total reads obtained from *Ancorina alata*, *Stellata maori*, *Stellata aremaria* sampled in New Zealand and *Xestospongia exigua* sampled on the Great Barrier Reef*.* In *X. exigua*, one low abundance OTU (0.17% of all reads) had 100% nucleotide identity with the V4 region of the *Ca*. N. ianthellae 16S rRNA gene. Among all environmental samples covered by the EMP, sequences highly similar or identical to *Ca*. N. ianthellae, with abundances above 0.1%, were exclusively found in sponges. Consistent with a habitat restricted to a few sponge species, no hits above 97.6% similarity to the 16S rRNA gene of *Ca*. N. ianthellae were detected by the Integrated Microbial NGS platform that queries most publicly available 16S rRNA gene amplicon data sets (but not the SMP dataset) (Lagkouvardos *et al*., 2016).

**CO_2_-fixation by *Ca.* N. ianthellae*.*** As expected, *Ca.* N. ianthellae encodes all key enzymes of the thaumarchaeal 3-hydroxypropionate/4-hydroxybutyrate pathway (Könneke *et al*., 2014; Otte *et al*., 2015) and three proteins catalyzing five steps of this thaumarchaeal CO_2_ fixation pathway were also detected within the metaproteome (Supporting Information Table S3).

**Lack of archaellum genes in *Ca.* N. ianthellae.** In contrast to several free-living AOA (Blainey *et al*., 2011; Jung *et al.*, 2014; Mosier *et al*., 2012; Spang *et al*., 2012; Bayer *et al*., 2016) and the DSGS-AOA, *Ca.* N. ianthellae, CCThau, and *Ca* C. symbiosum do not encode an archaellum. Lack of chemotaxis and motility traits has been described for other microbial symbionts (Karimi *et al*., 2018) and could represent an adaptation to the sponge-associated lifestyle, although also some non-sponge associated AOA lack an archaellum. The absence of an archaellum in *Ca.* N. ianthellae would also be consistent with vertical transmission of this symbiont, although this remains to be demonstrated as soon as larvae of *I. basta* will become available for experimentation.

**Eukaryotic-like proteins (ELPs) in** ***Ca.* Nitrosospongia ianthellae**. Four types of ELPs are found in *Ca*. N. ianthellae: Proteins with tetratricopeptide repeats (TPR), the Toll-interleukin-1 receptor (TIR) -like domain PF08937 (DUF1863; Cort *et al*., 2000), immunoglobulin-like (Ig-like) domains (DUF5011; Shigeno-Nakazawa *et al*., 2016), and hyaline repeats (HYR; Callebaut *et al*., 2000). The TPR were enriched in *Ca*. N. ianthellae when compared to other genome sequenced thaumarchaeotes, while the TIR and Ig-like domains were exclusive to *Ca*. N. ianthellae (Fig. 3, Supporting Information Fig. S6). Of the TPR containing proteins in *Ca*. N. ianthellae, 41 represent TPR gene families not previously detected in other thaumarchaeotes. TPR-containing proteins, which mediate protein-protein interactions in eukaryotes (Blatch and Lässle, 1999), are thought to be important for the survival of sponge symbionts in their phagocytic hosts. Two TPR-containing proteins from sponge microbiomes cloned into *E. coli* affected amoeba phagocytosis (Reynolds and Thomas, 2016), however, both proteins contained the TPR-Sel1 motif which was absent in the TPR-containing proteins from *Ca*. N. ianthellae. TIR-like proteins have also been found in other marine sponges (Wiens *et al*., 2006; Gauthier *et al*., 2010), with these proteins known to be key mediators of the metazoan innate immune response as well as playing a role in regulating metabolic and bioenergetic pathways through modulating NAD^+^ levels (Essuman *et al*., 2018). TIR-like proteins were expressed when sponges were subjected to bacteria-analogue lipoproteins (Wiens *et al*., 2006) and lipopolysaccharides (Wiens *et al*., 2005), which in turn caused the expression of a caspase likely involved in apoptosis and a macrophage-expressed protein, respectively. In this context, it is interesting to note that a protein encoding the DUF1863 domain from a zoonotic *Staphylococcus aureus* can decrease the survivability of mice infected by this strain (Patterson *et al*., 2014). Furthermore, the DUF1863 domain was recently shown to be a critical component of a bacterial defense system against myophages and may be involved in recognizing specific phage patterns (Doron *et al*., 2018).

An evolutionary homology between choanoflagellates and sponge choanocytes has long been speculated (Maldonado, 2005; Mah *et al*., 2014; Laundon et al., 2018). Both have diverse and abundant receptor tyrosine kinases (RTKs) (Srivastava *et al*., 2010; Miller, 2012), which are crucial components of metazoan signal transduction systems. Interestingly, choanoflagellate proteins with HYR-like domains were recently predicted to act as receptor tyrosine kinases (RTKs) (Manning *et al*., 2008). As HYR domains are structurally related to Ig and FN3 domains (Callebaut *et al*., 2000) and choanoflagellates lack the Ig domains found in many metazoan RTKs, the HYR domains may be fulfilling the role of the Ig domains in metazoan RTKs. Furthermore, the Ig-like DUF5011 domain has been found on the extracellular portion of two choanoflagellate homologues of the tyrosine kinase substrate BCAR1 (Shigeno-Nakazawa *et al*., 2016). Consequently, the numerous DUF5011 and HYR domain containing proteins of *Ca.* N. ianthellae may be interacting with the host signaling network as *I. basta* likely harbors similar extracellular domains as part of its signal transduction and gene regulatory processes. This host-symbiont interaction is further supported by observations that (i) many *Ca.* N. ianthellae proteins encoding these domains are predicted to be exported and (ii) five extracellular proteins of the *Ca.* N. ianthellae exclusive gene family containing the DUF5011 domain were detected in the metaproteome (Supporting Information Table S3). On the other hand it is noteworthy that regarding ELPs the *Ca.* N. ianthellae genome is lacking the ankyrin repeat proteins, leucine-rich repeat proteins, protein tyrosine kinases, and armadillo-repeat proteins frequently detected in other symbiotic and pathogenic microbes (Fan *et al*., 2012; Jernigan and Bordenstein, 2015).

**Supporting Experimental Procedures**

**Phylogenetic analyses.** For the *amoA* gene tree, Bayesian trees were constructed using Phylobayes v 4.1c (Lartillot and Philippe, 2004) using the best model identified for each dataset by ModelFinder (Kalyaanmoorthy *et al*., 2017). *Ca*. N. ianthellae and *Ca.* C. symbiosum *amoA* sequences were placed into a reference tree, representing all OTU representatives of the curated database provided in Alves *et al*. (2018), using the Evolutionary Placement Algorithm (EPA; Berger *et al*., 2011) implemented in RAxML-HPC 8.2.11 (Stamatakis, 2014). Representative sequences clustering with the *Ca*. N. ianthellae *amoA* gene sequence along with *amoA* sequences from the aforementioned genome-sequenced thaumarchaeota were then aligned with MUSCLE (Edgar, 2004) and analyzed

S-layer proteins were identified in all sequenced thaumarchaeota based on orthologous groups identified with Orthofinder containing members previously identified as SLPs (Li *et al*., 2018), or also classified as arCOG08647 in EggNOG version 4.5 (Huerta-Cepas *et al*., 2016). A phylogenetic reconstruction was performed with a thaumarchaeal-specific dataset with a minimal sequence length of 300 amino acids. Sequences were aligned using mafft (Katoh and Standley, 2013) and automatically trimmed using trimAl version 1.4 (Capella-Gutiérrez *et al*., 2009) and the -gappyout function. After model selection using ModelFinder (Kalyaanmoorthy *et al*., 2017) and a maximum-likelihood amino acid phylogenetic tree was generated using the model LG+F+G4 in IQ-Tree, version 1.6.2 (Nguyen *et al*., 2015) with 1,000 ultrafast bootstraps (UFBoot).

Genes encoding S08A family endopeptidases were identified in all sequenced thaumarchaeota if they contained the PF00082 (Peptidase_S8) domain when searched against the Pfam-A database. The complete sequence set was identified using thaumarchaeal amino acid sequences as individual queries for blastp searches against the Genbank nr database and only top hits containing the PF00082 domain were included. In total 277 representative sequences of the S08A family endopeptidases were used for phylogenetic analyses and sequences were trimmed according to the presence of the PF00082 domain before alignment using mafft (Katoh and Standley, 2013). ModelFinder (Kalyaanmoorthy *et al*., 2017) was used for model selection and maximum-likelihood phylogenetic analyses implemented with IQ-Tree, version 1.6.2 (Nguyen *et al*., 2015), using the LG+I+G4 model and 1,000 ultrafast bootstraps (UFBoot). Phylogenetic analyses for serpins (PF00079) were conducted in a similar fashion and 75 representative sequences were analyzed (after filtering out 46 distant homologues) using the WAG+I+G4 model and 1,000 ultrafast bootstraps.

Phylogenetic analyses were conducted on amino acid sequences of the *livK* periplasmic branched chain amino acid transporter subunit as well as a concatenated alignment of the *livFGHMK* operon, using the best model identified for each dataset by ModelFinder (Kalyaanmoorthy *et al*., 2017) and implemented in IQ-Tree, version 1.6.2 (Nguyen *et al*., 2015) with 1,000 ultrafast bootstraps (UFBoot). The complete sequence set was identified using individual *Ca*. N. ianthellae *livFGHMK* operon subunits as individual queries for blastp searches against the Genbank nr database. Top hits were included along with additional sequences demonstrated to be functional for active substrate transport within the hydrophobic amino-acid uptake transporter (HAAT) family (TCDB:3.A.14; Saier *et al*., 2009). Sequences were aligned using mafft (Katoh and Standley, 2013) and automatically trimmed using trimAl version 1.4 (Capella-Gutiérrez *et al*., 2009) and the -gappyout function. Models used for maximum-likelihood phylogenetic analyses were LG+F+G4 and LG+F+I+G4 for the *livK* subunit and the concatenated *livFGHMK* operon, respectively.

**Database screening for 16S rRNA gene amplicons.** A reference set of 16S rRNA gene sequences (N=65) from sequenced thaumarchaeotal genomes and amplicons associated with sponges, including *Ca.* N. bastadensis were aligned with SINA (Pruesse *et al*., 2012) and used to construct a reference tree in RaXML (Stamatakis, 2014). Sequence tags identified by the Sponge Microbiome Project (Moitinho-Silva *et al*., 2017b) were mapped to the reference set using blastn (Camacho *et al*., 2009), requiring at least 70% alignment and 90% identity. Successfully mapped reads were then aligned to the reference alignment using SINA and placed into the reference tree using RaXML-EPA (Berger *et al*, 2011). In addition, 16S rRNA gene sequences related to *Ca.* N. bastadensis were identified in short read archive (SRA) datasets using IMNGS (www.imngs.org - Lagkouvardos *et al*., 2016) with default parameters.

**
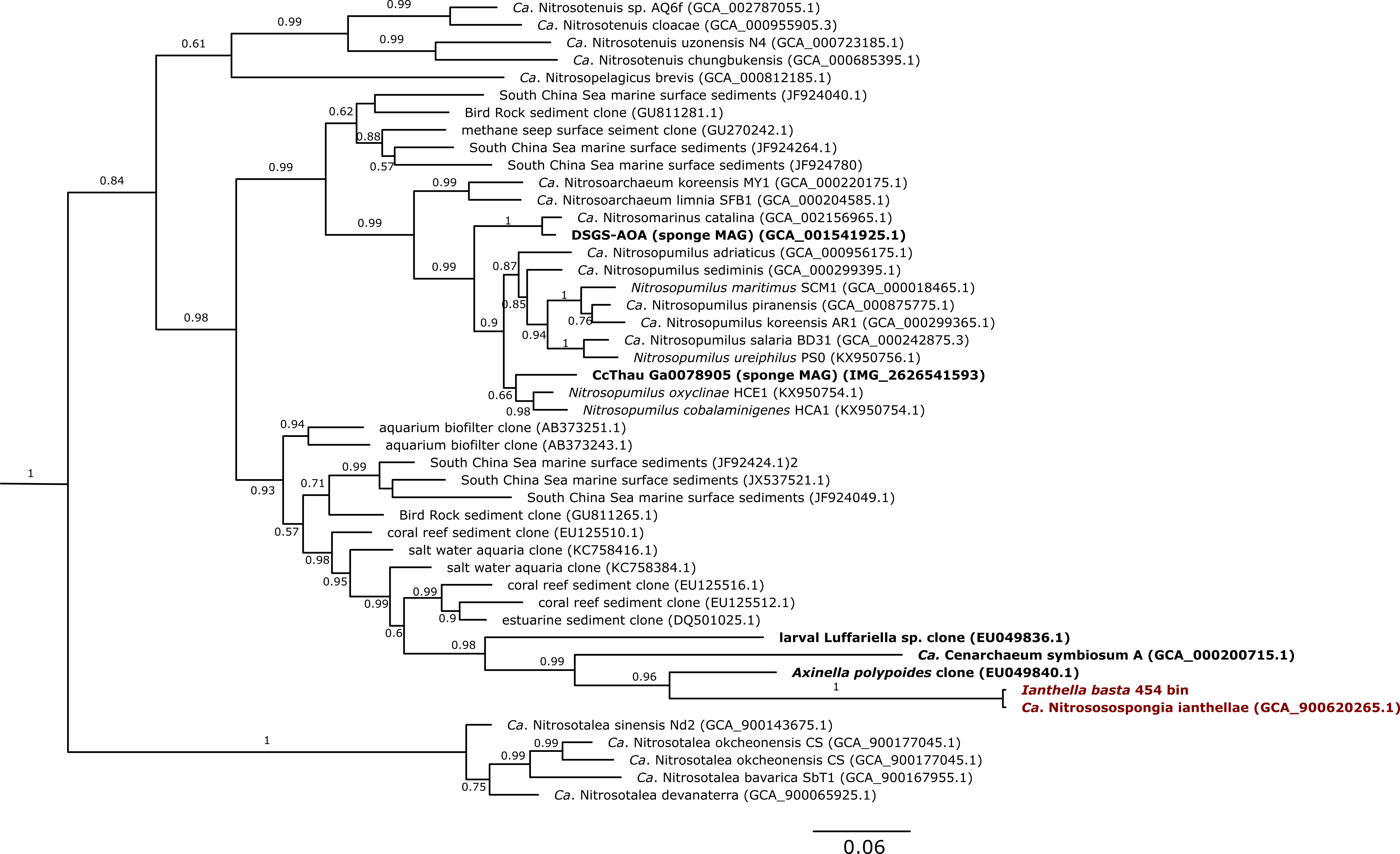
**

**Figure S1**. Bayesian *amoA* gene tree. For this analysis, *Ca*. N. ianthellae and *Ca.* C. symbiosum *amoA* sequences were placed into a reference tree, representing all OTU representatives of the curated database provided in Alves *et al.* (2018), using the Evolutionary Placement Algorithm (EPA; Berger *et al*., 2011) implemented in RAxML-HPC 8.2.11 (Stamatakis, 2014). Representative sequences clustering with the sponge symbiont *amoA* sequences were then analyzed along with *amoA* sequences from the other genome-sequenced AOA. Note that *Ca.* Nitrosotalea okcheonensis possesses two *amoA* gene copies (Herbold *et al*., 2017). Outgroups for both trees consisted of all three genome-sequenced members of the *Nitrososphaera* cluster, both members of the *Nitrosocosmicus* clade, and *Ca.* Nitrosocaldus icelandicus. In all trees, sequences obtained from sponges are depicted in bold.

**
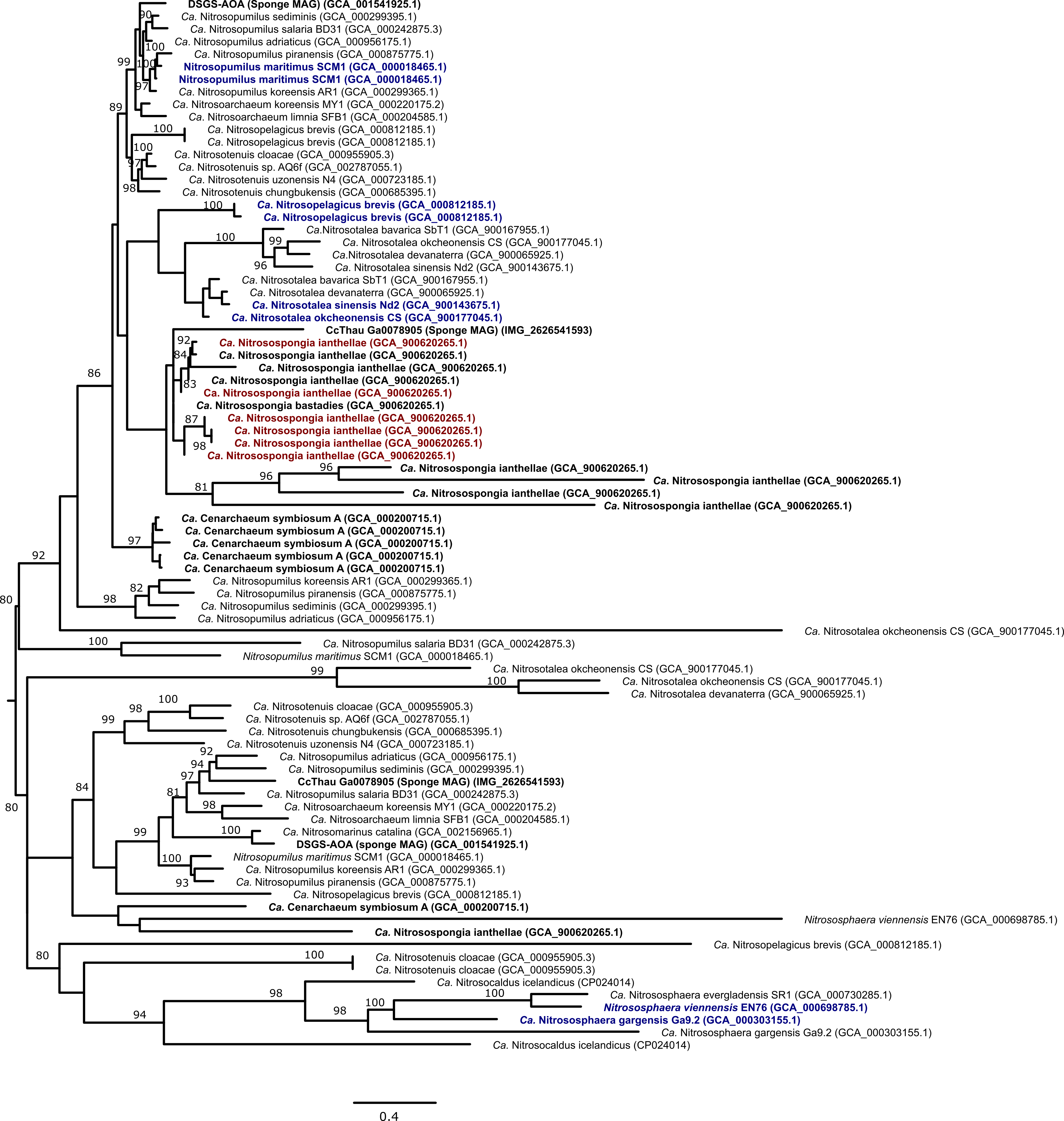
**

**Figure S2**. Maximum-likelihood amino acid phylogenetic tree (after automatic model selection with IQ-Tree, version 1.6.2) of putative S-layer proteins found in all analyzed thaumarchaeal genomes (28 in total). Sponge-derived sequences are depicted in bold black, while highly expressed proteins from *Ca*. N. ianthellae are depicted in bold red (6 in total). Highlighted in bold blue are putative S-layer proteins found to be highly expressed in previous proteomics studies (Santoro *et al*., 2015; Palatinszky *et al*., 2015; Kerou *et al*., 2016; Qin *et al*., 2017; Herbold *et al*., 2017). Values at nodes represent ultrafast bootstraps (UFBoot) with only values ≥80% shown for each branch.

**
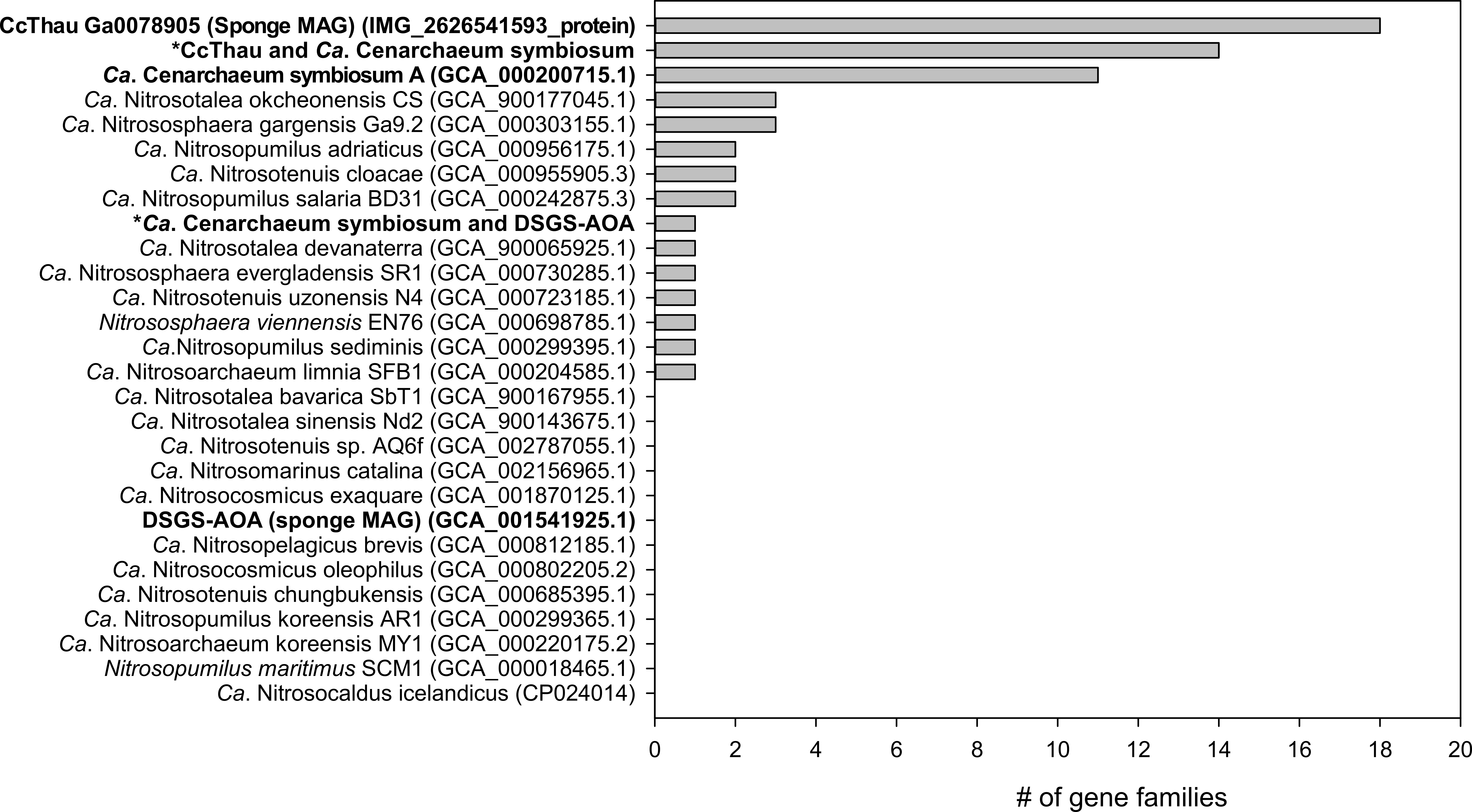
**

**Figure S3.** Number of gene families shared exclusively between *Ca*. N. ianthellae and each genome-sequenced member of the *Thaumarchaeota* including the sponge thaumarchaeal symbionts, CCThau, *Ca*. C. symbiosum, and the DSGS-AOA (in bold). (*) indicates gene families shared by *Ca*. N. ianthellae with *Ca.* C. symbiosum and CcThau exclusively, or with *Ca*. C. symbiosum and the glass sponge AOA exclusively.

**

**

**Figure S4.** Maximum-likelihood amino acid phylogenetic trees (after automatic model selection with IQ-Tree, version 1.6.2) of (A) S08A family endopeptidases and (B) serine protease inhibitors (serpins). Color coding donates the degree of homology among all sequenced *Thaumarchaeota*: light blue – shared exclusively among thaumarchaeal sponge symbionts; dark blue – ubiquitously found in *Thaumarchaeota*. In (A), 6 out of 8 *Ca.* N. ianthellae S08A family endopeptidases, within the “Marine sponge Thaumarchaeota subtilisin” clade, are predicted to be exported. On the other hand, the majority of the ubiquitous “Unclassified archaea subtilisin 2” (24/27) and all except one of the “MG-1 like subtilisin” (naming convention after Li *et al*., 2015) are predicted to be membrane anchored S08A endopeptidases. In (B), 3 of the 15 serpins found in *Ca*. N. ianthellae were excluded from phylogenetic analyses due to truncated length. Values at nodes represent ultrafast bootstraps (UFBoot) with only values ≥80% shown for each branch.

**
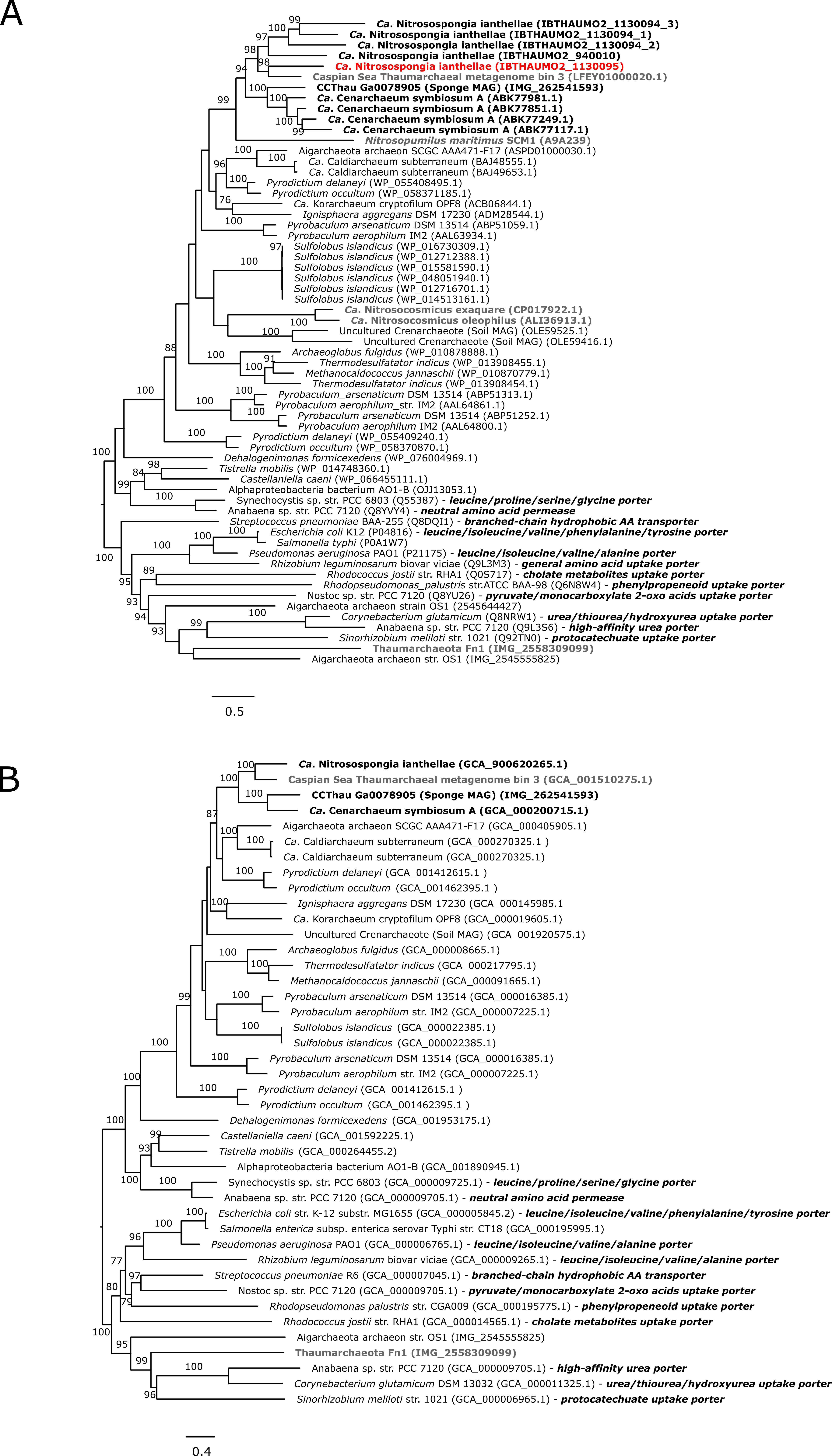
**

**Figure S5**. Maximum-likelihood amino acid phylogenetic trees (after automatic model selection with IQ-Tree, version 1.6.2) for the periplasmic subunit (A) *LivK* and for the concatenated (B) *LivFGHMK* operon. *LivK* was found to be highly expressed (expressed copy in red) in the *Ca*. N. ianthellae proteome and can occur in multiple copies among *livFGHMK* encoding microorganisms. *Ca*. N. ianthellae has two *livK* genes and a gene containing three fused *LivK* domains and the predicted proteins from these five *livK* genes/domains were included in the phylogenetic analysis. *LivK* is also found in some other thaumarchaeotes (bold grey), but these lack the *livFGHM* genes necessary for branched-chain amino acid transport. In a few cases in the concatenated *livFGHMK* tree, multiple operons were present in a given genome. For both trees, sponge thaumarchaeal sequences are highlighted in bold black while other thaumarchaeal sequences are highlighted in bold grey. Sequences from organisms where the specific transport functions have been identified, have those specific functions annotated. These annotations are taken from information collated in the Transporter Classification database (Saier *et al*., 2009). Values at nodes represent ultrafast bootstraps (UFBoot) with only values ≥80% shown for each branch.


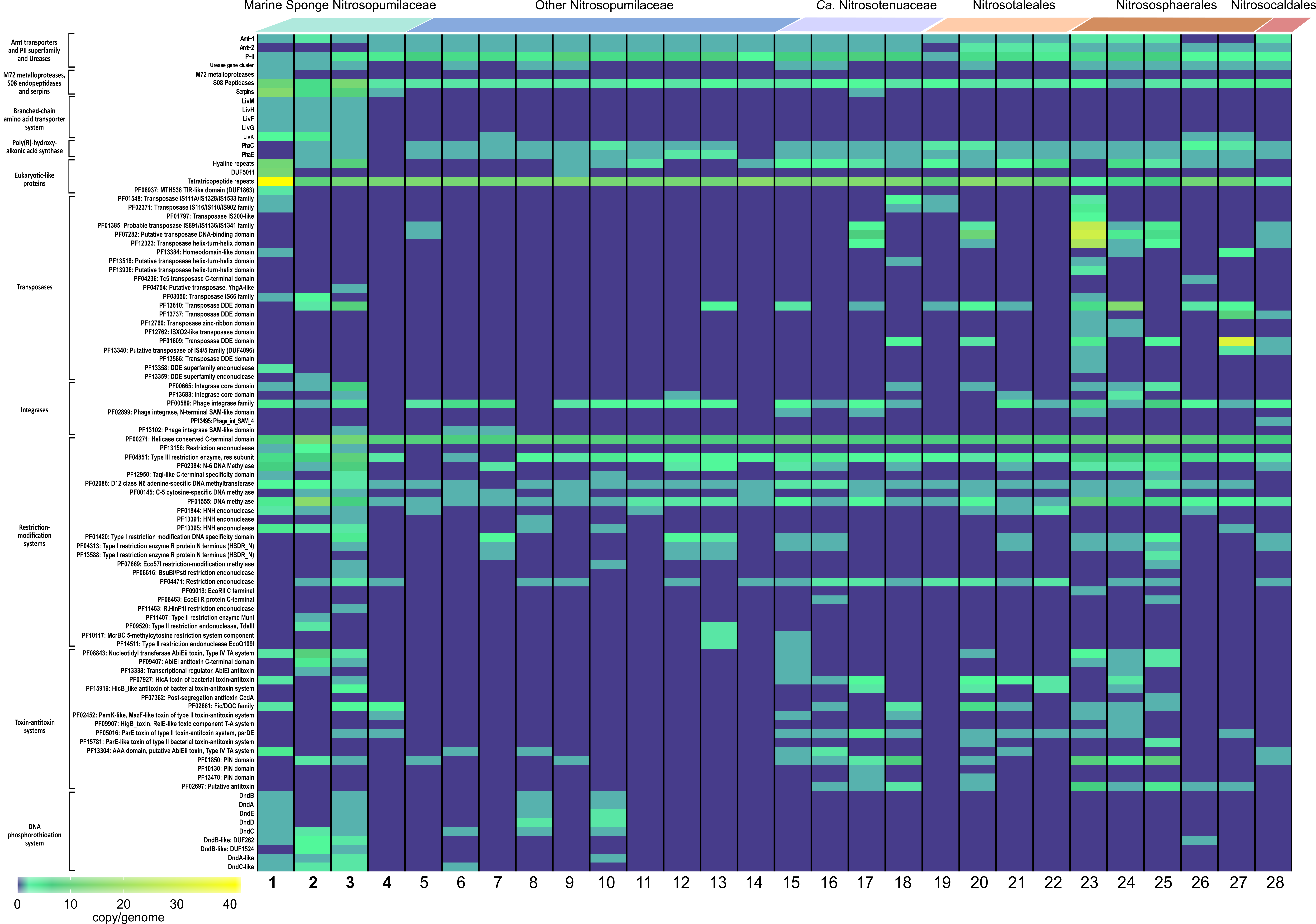


**Figure S6.** Heat map showing the distribution and gene copy number per genome of selected genes, gene classes and PFAM annotations among genome-sequenced AOA. The color scale ranges from 0 (dark blue) to 40 (yellow) and indicates copies per genome. Sponge-derived genomes start on the left and are depicted in bold, followed by members of *Ca*. Nitrosopumilaceae, *Ca*. Nitrosotenuaceae, *Ca*. Nitrosotaleale, the *Nitrososphaerales*, and *Ca*. Nitrosocaldales, respectively. Numbers at the bottom of the figure refer to the AOA genomes: **1)** *Ca*. N. ianthellae, **2)** *Ca*. C. symbiosum, **3)** CcThau Ga007890, **4)** DSGS-AOA, **5)** *Ca*. N. koreensis AR1, **6)** *Ca*. N. piranensis, **7)** *N. maritimus* SCM1, **8)** *Ca*. N. catalina, **9)** *Ca*. N. sediminis, **10)** *Ca*. N. salaria, **11)** *Ca*. N. adriaticus, **12)** *Ca*. N. koreensis MY1, **13)** N. limnia SFB1, **14)** *Ca*. N. brevis, **15)** *Ca*. N sp. AQ6f, **16)** *Ca*. N. cloacae, **17)** *Ca*. N. chungbukensis, **18)** *Ca*. N. uzonensis, **19)** *Ca*. N. bavarica, **20)** *Ca*. N. okcheonensis, **21)** *Ca*. N. devanaterra, **22)** *Ca*. N. siniensis, **23)** *Ca*. N. gargensis, **24)** *N. viennensis*, **25)** *Ca*. N. evergladensis, **26)** *Ca*. N. exaquare, **27)** *Ca*. N. oleophilus, and **28)** *Ca*. N. icelandicus.

**
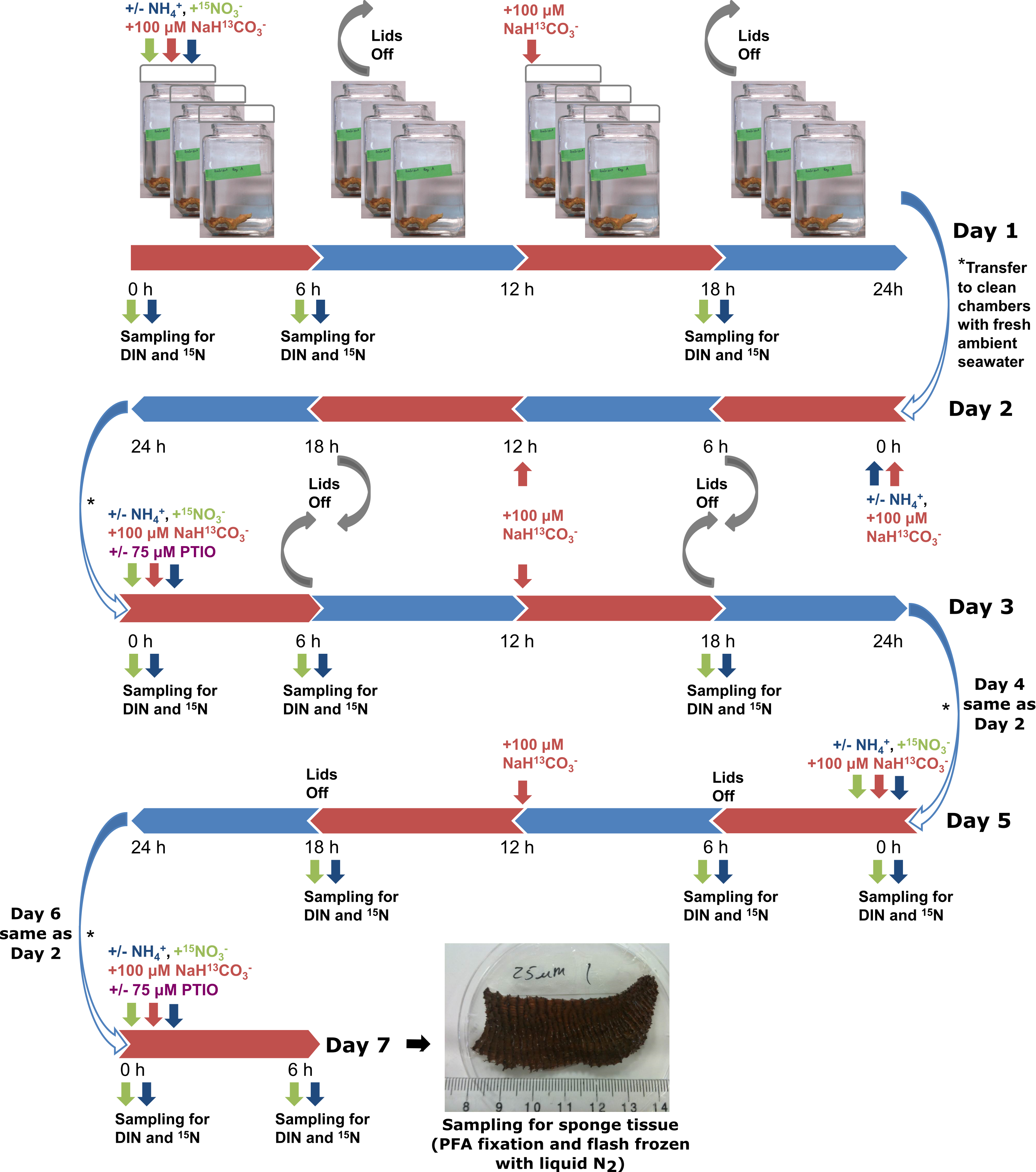
**

**Figure S7.** Experimental design for the *I. basta* holobiont nitrification incubations. The intermittently closed setup was employed to avoid oxygen depletion while minimizing loss of ^13^C-labeled HCO_3_^-^. Green and blue arrows denote samples used for the calculation of net and gross nitrification rates, respectively. Days 4, 5 and 6 were recovery days for those incubations to which PTIO was added.

**
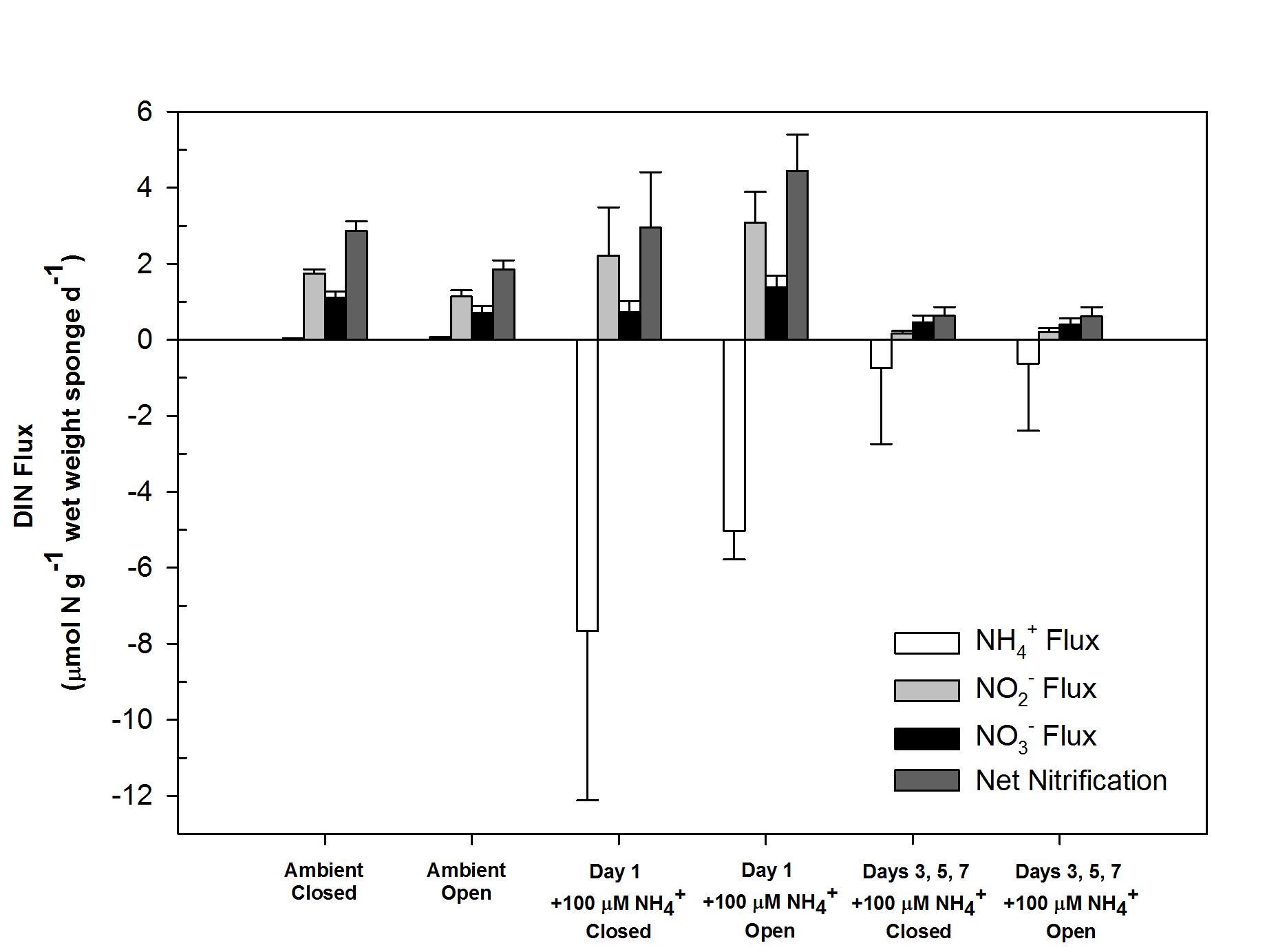
**

**Figure S8.** Comparison of average DIN (NH_4_^+^, NO_3_^-^, NO_2_^-^) flux and net nitrification rates exhibited by the *I. basta* holobiont in multiple-day incubations between intermittently closed (Supporting Information Figure 1) and completely open aquaria at ambient conditions and at 100 μM NH_4_^+^. Pair-wise comparisons of individual DIN species flux and net nitrification between open and intermittently closed aquaria, revealed significant differences in net NO_2_^-^ flux and net nitrification between ambient treatments (both: *p* < 0.01, Mann-Whitney U-test).


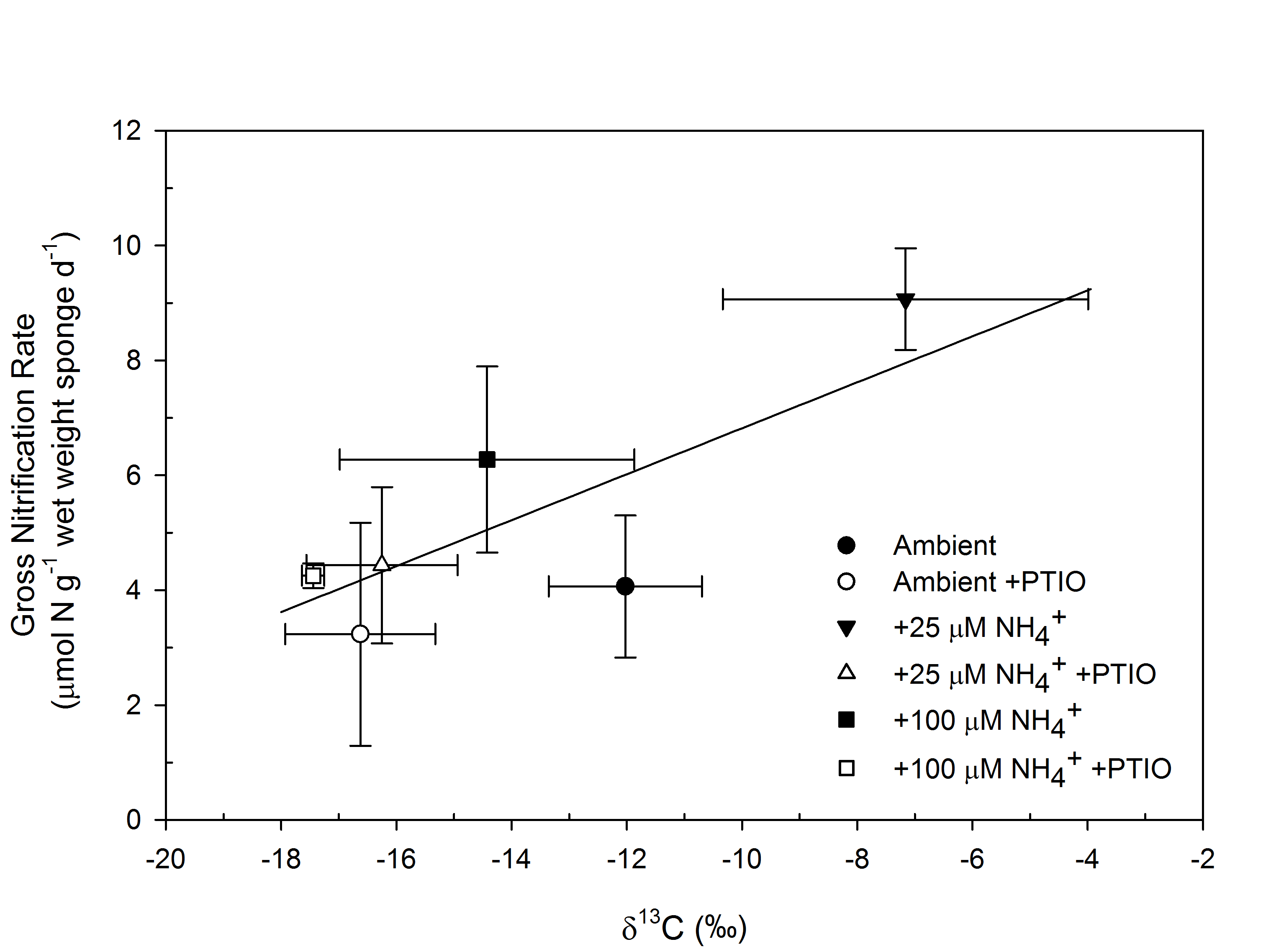


**Figure S9.** Nitrification activity of the *I. basta* holobiont during 7-day incubation experiments at ambient conditions and with added ammonium (25 or 100 µM) and with/without the AOA inhibitor PTIO. The relationship between δ^13^C values of sponge clones (labeled from added ^13^C-bicarbonate) sampled on day 7 and gross nitrification rates of the *I. basta* holobiont is depicted. To yield average nitrification activity over the course of the incubation, we used the weighted mean of the gross nitrification rates (i.e., days are weights). For comparison, the δ^13^C value reflecting the natural abundance was determined from the sponge individual used for Illumina metagenome sequencing and found to be -21.7 ‰ (not shown). The δ^13^C values derived from the *I. basta* holobiont nitrification experiments displayed a positive and significant correlation with gross nitrification rates (R = 0.656, *p* < 0.005). Error bars reflect the standard error of the sample mean, where n = 3 for the δ^13^C values and for all weighted gross nitrification rates in all treatment conditions. PTIO additions started two days after the experiment was commenced; hence fixation of ^13^C-labeled bicarbonate during the first two days was not influenced by inhibition.

| **Bin ID** | **IBThaumO2** |
| --- | --- |
| Analysis project type | metagenome-assembled genome (MAG) |
| Taxa_id | 16S rRNA and multi-marker phylogenetics |
| Assembly software | Spades |
| Annotation | MaGe |
| Genome Quality | High Quality Draft |
| Completeness (%) | 99.03 |
| Contamination (%) | 0.97 |
| Completeness/Contamination Software | CheckM |
| Number of contigs | 113 |
| 16S rRNA gene recovered | yes |
| 16S rRNA gene recovery software | Rnammer / MaGe |
| Number of standard tRNAs extracted | 20 |
| tRNA extraction software | tRNA-scan / MaGe |
| Binning software | metabat2 |
| Binning parameters | kmer |
| Genome size (Mbp) | 1.99 |
| N50 (bp) | 35099 |
| Longest contig (bp) | 135,585 |
| Average contig length (bp) | 17,669.70 |
| GC content (%) | 64.8 |
| Protein coding sequences | 2,342 |
| rRNAs | 3 |
| tRNAs | 42 |

**Table S1**. Overview of genome binning parameters, statistics and associated metadata for the *Ca.* N. ianthellae genome bin.

| **Table S2.** Presence of genes of interest (see Figure 3) in the two*Ca.* N. ianthellae MAGs (obtained from two*I. basta* individuals by Illumina and 454 pyrosequencing, respectively) as determined by BLASTp. | | | | | | | | | | |
| --- | --- | --- | --- | --- | --- | --- | --- | --- | --- | --- |
|  | qseqid | sseqid | pident | length | qlen | slen | evalue | bitscore | query match coverage | subject match coverage |
| M72 metalloproteases | IBTHAUMO2_880008 | IBTHAUMv1_16080007 | 64.242 | 660 | 2028 | 2440 | 0 | 729 | 32.54 | 26.76 |
| S08 Peptidases | IBTHAUMO2_1030012 | IBTHAUMv1_12290002 | 91.578 | 5141 | 5956 | 6201 | 0 | 9250 | 86.32 | 82.26 |
|  | IBTHAUMO2_150018 | IBTHAUMv1_17130004 | 99.286 | 560 | 908 | 611 | 0 | 1133 | 61.67 | 91.49 |
|  | IBTHAUMO2_160002 | IBTHAUMv1_1950001 | 100 | 659 | 1181 | 1349 | 0 | 1355 | 55.80 | 48.78 |
|  | IBTHAUMO2_170054 | IBTHAUMv1_3990003 | 99.646 | 1695 | 1695 | 1695 | 0 | 3440 | 100.00 | 99.94 |
|  | IBTHAUMO2_220007 | IBTHAUMv1_13830026 | 99.542 | 655 | 655 | 655 | 0 | 1257 | 100.00 | 99.85 |
|  | IBTHAUMO2_250004 | IBTHAUMv1_4210010 | 65.672 | 268 | 303 | 1619 | 1.16E-98 | 315 | 88.45 | 16.24 |
|  | IBTHAUMO2_440007 | IBTHAUMv1_14780018 | 99.856 | 696 | 699 | 696 | 0 | 1429 | 99.57 | 99.86 |
|  | IBTHAUMO2_470031 | IBTHAUMv1_15480003 | 99.794 | 1459 | 1459 | 1459 | 0 | 2938 | 100.00 | 99.93 |
|  | IBTHAUMO2_470037 | IBTHAUMv1_15480010 | 99.446 | 1264 | 1264 | 1266 | 0 | 2486 | 100.00 | 99.76 |
|  | IBTHAUMO2_600001 | IBTHAUMv1_1950001 | 89.893 | 1029 | 1563 | 1349 | 0 | 1843 | 65.83 | 76.20 |
|  | IBTHAUMO2_810001 | IBTHAUMv1_16080007 | 92.872 | 2413 | 2385 | 2440 | 0 | 4403 | 101.17 | 98.11 |
| Serpins | IBTHAUMO2_1040003 | IBTHAUMv1_16780011 | 78.208 | 413 | 472 | 412 | 0 | 620 | 87.50 | 99.76 |
|  | IBTHAUMO2_150002 | IBTHAUMv1_9590002 | 100 | 412 | 412 | 412 | 0 | 835 | 100.00 | 99.76 |
|  | IBTHAUMO2_150006 | IBTHAUMv1_2680003 | 99.589 | 487 | 487 | 487 | 0 | 979 | 100.00 | 99.79 |
|  | IBTHAUMO2_150019 | IBTHAUMv1_17130003 | 100 | 468 | 501 | 499 | 0 | 943 | 93.41 | 93.59 |
|  | IBTHAUMO2_160001 | IBTHAUMv1_15480013 | 98.14 | 645 | 646 | 649 | 0 | 1268 | 99.85 | 99.23 |
|  | IBTHAUMO2_250003 | IBTHAUMv1_9590002 | 83.846 | 390 | 413 | 412 | 0 | 665 | 94.43 | 92.48 |
|  | IBTHAUMO2_250005 | IBTHAUMv1_3270002 | 50.427 | 117 | 107 | 377 | 4.53E-25 | 94.4 | 109.35 | 30.77 |
|  | IBTHAUMO2_470041 | IBTHAUMv1_17030009 | 92.308 | 273 | 389 | 649 | 0 | 521 | 70.18 | 41.91 |
|  | IBTHAUMO2_470042 | IBTHAUMv1_16080008 | 74.297 | 498 | 498 | 498 | 0 | 667 | 100.00 | 99.20 |
|  | IBTHAUMO2_530010 | IBTHAUMv1_3270002 | 97.706 | 218 | 240 | 377 | 3.43E-150 | 421 | 90.83 | 57.56 |
|  | IBTHAUMO2_530011 | IBTHAUMv1_3270002 | 98.052 | 154 | 154 | 377 | 7.63E-104 | 299 | 100.00 | 40.58 |
|  | IBTHAUMO2_630004 | IBTHAUMv1_4530014 | 100 | 419 | 419 | 419 | 0 | 853 | 100.00 | 99.76 |
|  | IBTHAUMO2_740001 | IBTHAUMv1_2680003 | 95.833 | 72 | 74 | 487 | 2.60E-36 | 124 | 97.30 | 14.58 |
|  | IBTHAUMO2_810003 | IBTHAUMv1_16080004 | 100 | 474 | 474 | 474 | 0 | 970 | 100.00 | 99.79 |
|  | IBTHAUMO2_880009 | IBTHAUMv1_16080008 | 100 | 498 | 498 | 498 | 0 | 1014 | 100.00 | 99.80 |
| LivM | IBTHAUMO2_1130089 | IBTHAUMv1_13040008 | 99.717 | 353 | 353 | 353 | 0 | 677 | 100.00 | 99.72 |
| LivH | IBTHAUMO2_1130090 | IBTHAUMv1_13040007 | 100 | 306 | 306 | 306 | 0 | 587 | 100.00 | 99.67 |
| LivF | IBTHAUMO2_1130093 | IBTHAUMv1_13040004 | 100 | 238 | 238 | 238 | 1.80E-172 | 471 | 100.00 | 99.58 |
| LivG | IBTHAUMO2_1130092 | IBTHAUMv1_13040005 | 99.209 | 253 | 269 | 253 | 0 | 496 | 94.05 | 99.60 |
| LivK | IBTHAUMO2_1130094 | IBTHAUMv1_13040003 | 99.688 | 642 | 1149 | 642 | 0 | 1236 | 55.87 | 99.84 |
| LivK | IBTHAUMO2_1130095 | IBTHAUMv1_16960002 | 93.738 | 527 | 527 | 511 | 0 | 993 | 100.00 | 99.80 |
| LivK | IBTHAUMO2_940010 | IBTHAUMv1_330001 | 100 | 314 | 402 | 314 | 0 | 622 | 78.11 | 99.68 |
| DndB | IBTHAUMO2_810011 | IBTHAUMv1_190016 | 44.688 | 320 | 362 | 319 | 2.21E-98 | 292 | 88.40 | 98.43 |
| DndA | IBTHAUMO2_880013 | IBTHAUMv1_140010 | 70.712 | 379 | 391 | 388 | 0 | 566 | 96.93 | 97.42 |
| DndE | IBTHAUMO2_880014 | IBTHAUMv1_140009 | 39.655 | 116 | 123 | 126 | 1.18E-32 | 108 | 94.31 | 91.27 |
| DndD | IBTHAUMO2_880015 | IBTHAUMv1_190014 | 34.074 | 675 | 675 | 660 | 1.80E-107 | 337 | 100.00 | 99.55 |
| DndC | IBTHAUMO2_880016 | IBTHAUMv1_140006 | 56.592 | 493 | 473 | 502 | 0 | 576 | 104.23 | 98.01 |
| DndB-like: DUF262 | IBTHAUMO2_1050033 | IBTHAUMv1_380002 | 27.711 | 166 | 425 | 344 | 2.67E-10 | 58.9 | 39.06 | 44.48 |
| DndA-like | IBTHAUMO2_690025 | IBTHAUMv1_3820004 | 99.482 | 386 | 386 | 386 | 0 | 772 | 100.00 | 99.74 |
| DndC-like | IBTHAUMO2_690027 | IBTHAUMv1_17710003 | 100 | 280 | 280 | 280 | 0 | 582 | 100.00 | 99.64 |
| PF08937: MTH538 TIR-like | IBTHAUMO2_460006 | IBTHAUMv1_14780023 | 100 | 130 | 130 | 170 | 7.13E-94 | 265 | 100.00 | 75.88 |
|  | IBTHAUMO2_620008 | IBTHAUMv1_14750015 | 100 | 172 | 172 | 172 | 3.99E-126 | 349 | 100.00 | 99.42 |
| Hyaline repeats | IBTHAUMO2_150018 | IBTHAUMv1_17130004 | 99.286 | 560 | 908 | 611 | 0 | 1133 | 61.67 | 91.49 |
|  | IBTHAUMO2_1050019 | IBTHAUMv1_14910004 | 99.774 | 442 | 442 | 442 | 0 | 880 | 100.00 | 99.77 |
|  | IBTHAUMO2_1050020 | IBTHAUMv1_14910005 | 75.036 | 701 | 705 | 702 | 0 | 988 | 99.43 | 99.15 |
|  | IBTHAUMO2_1110002 | IBTHAUMv1_460002 | 44.798 | 471 | 491 | 487 | 8.48E-124 | 368 | 95.93 | 96.51 |
|  | IBTHAUMO2_1110003 | IBTHAUMv1_15140009 | 49.099 | 444 | 443 | 452 | 2.55E-124 | 366 | 100.23 | 97.35 |
|  | IBTHAUMO2_220002 | IBTHAUMv1_150003 | 86.853 | 715 | 712 | 715 | 0 | 1217 | 100.42 | 99.86 |
|  | IBTHAUMO2_450016 | IBTHAUMv1_3920005 | 97.511 | 442 | 630 | 442 | 0 | 862 | 70.16 | 99.77 |
|  | IBTHAUMO2_470043 | IBTHAUMv1_16080007 | 92.531 | 241 | 237 | 2440 | 1.02E-125 | 389 | 101.69 | 9.80 |
|  | IBTHAUMO2_720042 | IBTHAUMv1_14780010 | 48.347 | 242 | 268 | 742 | 1.76E-63 | 209 | 90.30 | 31.81 |
|  | IBTHAUMO2_810001 | IBTHAUMv1_16080007 | 92.872 | 2413 | 2385 | 2440 | 0 | 4403 | 101.17 | 98.11 |
|  | IBTHAUMO2_880008 | IBTHAUMv1_16080007 | 64.242 | 660 | 2028 | 2440 | 0 | 729 | 32.54 | 26.76 |
| PF16403: DUF5011 | IBTHAUMO2_1030012 | IBTHAUMv1_12290002 | 91.578 | 5141 | 5956 | 6201 | 0 | 9250 | 86.32 | 82.26 |
|  | IBTHAUMO2_170054 | IBTHAUMv1_3990003 | 99.646 | 1695 | 1695 | 1695 | 0 | 3440 | 100.00 | 99.94 |
|  | IBTHAUMO2_20012 | IBTHAUMv1_11360007 | 95.513 | 936 | 1651 | 962 | 0 | 1722 | 56.69 | 97.19 |
|  | IBTHAUMO2_240080 | IBTHAUMv1_11360001 | 98.649 | 2073 | 2073 | 2073 | 0 | 4182 | 100.00 | 99.95 |
|  | IBTHAUMO2_290009 | IBTHAUMv1_13830036 | 99.644 | 1966 | 1966 | 1966 | 0 | 3962 | 100.00 | 99.95 |
|  | IBTHAUMO2_320050 | IBTHAUMv1_2050001 | 97.937 | 1115 | 1328 | 1117 | 0 | 2196 | 83.96 | 99.73 |
|  | IBTHAUMO2_460001 | IBTHAUMv1_14780028 | 91.978 | 1633 | 1773 | 4551 | 0 | 2982 | 92.10 | 35.62 |
|  | IBTHAUMO2_470031 | IBTHAUMv1_15480003 | 99.794 | 1459 | 1459 | 1459 | 0 | 2938 | 100.00 | 99.93 |
|  | IBTHAUMO2_590001 | IBTHAUMv1_20280001 | 98.699 | 615 | 648 | 1427 | 0 | 1256 | 94.91 | 43.03 |
|  | IBTHAUMO2_590078 | IBTHAUMv1_13120027 | 99.522 | 837 | 1040 | 848 | 0 | 1659 | 80.48 | 98.58 |
|  | IBTHAUMO2_620022 | IBTHAUMv1_12370012 | 98.862 | 1845 | 1845 | 2170 | 0 | 3714 | 100.00 | 84.98 |
|  | IBTHAUMO2_700001 | IBTHAUMv1_18570002 | 76.798 | 2836 | 3095 | 8426 | 0 | 4177 | 91.63 | 33.54 |
|  | IBTHAUMO2_990001 | IBTHAUMv1_4410003 | 99.731 | 1116 | 1116 | 11182 | 0 | 2271 | 100.00 | 9.97 |
| Tetratricopeptide repeats | IBTHAUMO2_20005 | IBTHAUMv1_4210007 | 100 | 542 | 542 | 561 | 0 | 1096 | 100.00 | 96.43 |
|  | IBTHAUMO2_260017 | IBTHAUMv1_1700002 | 99.743 | 389 | 389 | 394 | 0 | 786 | 100.00 | 98.48 |
|  | IBTHAUMO2_450009 | IBTHAUMv1_15960007 | 98.947 | 665 | 665 | 665 | 0 | 1304 | 100.00 | 99.85 |
|  | IBTHAUMO2_590071 | IBTHAUMv1_13120019 | 98.704 | 463 | 463 | 463 | 0 | 926 | 100.00 | 99.78 |
|  | IBTHAUMO2_690013 | IBTHAUMv1_11050005 | 100 | 236 | 236 | 236 | 4.75E-174 | 475 | 100.00 | 99.58 |
|  | IBTHAUMO2_250010 | IBTHAUMv1_1040006 | 97.196 | 214 | 214 | 214 | 2.07E-147 | 406 | 100.00 | 99.53 |
|  | IBTHAUMO2_590041 | IBTHAUMv1_4080009 | 98.78 | 738 | 738 | 738 | 0 | 1435 | 100.00 | 99.86 |
|  | IBTHAUMO2_730001 | IBTHAUMv1_5300001 | 97.778 | 90 | 123 | 144 | 3.63E-61 | 181 | 73.17 | 61.81 |
|  | IBTHAUMO2_1130054 | IBTHAUMv1_13940010 | 100 | 262 | 262 | 262 | 0 | 514 | 100.00 | 99.62 |
|  | IBTHAUMO2_10010 | IBTHAUMv1_1840004 | 33.738 | 412 | 416 | 754 | 3.80E-61 | 208 | 99.04 | 54.24 |
|  | IBTHAUMO2_1080005 | IBTHAUMv1_16190004 | 99.153 | 118 | 118 | 118 | 1.76E-83 | 237 | 100.00 | 99.15 |
|  | IBTHAUMO2_1120015 | IBTHAUMv1_5290001 | 59.541 | 566 | 738 | 565 | 0 | 629 | 76.69 | 99.47 |
|  | IBTHAUMO2_170044 | IBTHAUMv1_3990013 | 99.425 | 174 | 174 | 174 | 1.18E-117 | 328 | 100.00 | 99.43 |
|  | IBTHAUMO2_200001 | IBTHAUMv1_15480023 | 99.693 | 326 | 417 | 326 | 0 | 643 | 78.18 | 99.69 |
|  | IBTHAUMO2_20006 | IBTHAUMv1_4210006 | 100 | 495 | 495 | 495 | 0 | 997 | 100.00 | 99.80 |
|  | IBTHAUMO2_260040 | IBTHAUMv1_1140003 | 99.635 | 274 | 274 | 274 | 0 | 539 | 100.00 | 99.64 |
|  | IBTHAUMO2_330008 | IBTHAUMv1_17060002 | 94.872 | 429 | 457 | 443 | 0 | 805 | 93.87 | 96.61 |
|  | IBTHAUMO2_410008 | IBTHAUMv1_13120019 | 45.02 | 251 | 274 | 463 | 1.19E-66 | 212 | 91.61 | 52.92 |
|  | IBTHAUMO2_410009 | IBTHAUMv1_1610001 | 94.574 | 387 | 399 | 394 | 0 | 697 | 96.99 | 97.97 |
|  | IBTHAUMO2_440013 | IBTHAUMv1_14780004 | 86.288 | 598 | 598 | 598 | 0 | 1035 | 100.00 | 99.83 |
|  | IBTHAUMO2_450076 | IBTHAUMv1_15360002 | 100 | 299 | 299 | 299 | 0 | 563 | 100.00 | 99.67 |
|  | IBTHAUMO2_480001 | IBTHAUMv1_3180004 | 100 | 339 | 339 | 339 | 0 | 669 | 100.00 | 99.71 |
|  | IBTHAUMO2_480002 | IBTHAUMv1_3180005 | 99.76 | 416 | 434 | 416 | 0 | 793 | 95.85 | 99.76 |
|  | IBTHAUMO2_510001 | IBTHAUMv1_2270002 | 100 | 279 | 279 | 534 | 0 | 553 | 100.00 | 52.06 |
|  | IBTHAUMO2_690014 | IBTHAUMv1_11050004 | 98.305 | 236 | 236 | 236 | 9.73E-172 | 469 | 100.00 | 99.58 |
|  | IBTHAUMO2_650002 | IBTHAUMv1_20210002 | 100 | 262 | 262 | 262 | 0 | 516 | 100.00 | 99.62 |
|  | IBTHAUMO2_700005 | IBTHAUMv1_21200002 | 96.032 | 252 | 256 | 256 | 4.84E-171 | 469 | 98.44 | 98.05 |
|  | IBTHAUMO2_750012 | IBTHAUMv1_2270002 | 100 | 241 | 243 | 534 | 4.86E-176 | 492 | 99.18 | 44.94 |
|  | IBTHAUMO2_790015 | IBTHAUMv1_15480026 | 99.77 | 435 | 435 | 435 | 0 | 852 | 100.00 | 99.77 |
|  | IBTHAUMO2_880020 | IBTHAUMv1_2270002 | 99.17 | 241 | 268 | 534 | 1.46E-174 | 489 | 89.93 | 44.94 |
|  | IBTHAUMO2_940002 | IBTHAUMv1_4830003 | 96.811 | 439 | 439 | 439 | 0 | 838 | 100.00 | 99.77 |
|  | IBTHAUMO2_990018 | IBTHAUMv1_16760014 | 100 | 140 | 140 | 140 | 2.46E-92 | 261 | 100.00 | 99.29 |
|  | IBTHAUMO2_200002 | IBTHAUMv1_15480022 | 93.885 | 278 | 283 | 286 | 4.26E-178 | 489 | 98.23 | 96.85 |
|  | IBTHAUMO2_20010 | IBTHAUMv1_4210003 | 98.396 | 187 | 187 | 187 | 1.12E-126 | 352 | 100.00 | 99.47 |
|  | IBTHAUMO2_720008 | IBTHAUMv1_4130013 | 100 | 127 | 127 | 127 | 1.21E-88 | 250 | 100.00 | 99.21 |
|  | IBTHAUMO2_380029 | IBTHAUMv1_11240003 | 100 | 309 | 309 | 309 | 0 | 606 | 100.00 | 99.68 |
|  | IBTHAUMO2_590042 | IBTHAUMv1_4080008 | 99.286 | 420 | 423 | 423 | 0 | 845 | 99.29 | 99.05 |
|  | IBTHAUMO2_990017 | IBTHAUMv1_16760013 | 100 | 68 | 68 | 68 | 7.21E-43 | 130 | 100.00 | 98.53 |
|  | IBTHAUMO2_480009 | IBTHAUMv1_90003 | 100 | 197 | 197 | 237 | 5.88E-143 | 395 | 100.00 | 82.70 |
|  | IBTHAUMO2_700003 | IBTHAUMv1_20360001 | 100 | 204 | 204 | 252 | 2.91E-149 | 412 | 100.00 | 80.56 |
| Transposases | IBTHAUMO2_1050022 | IBTHAUMv1_8580062 | 99.457 | 368 | 368 | 368 | 0 | 733 | 100.00 | 99.73 |
|  | IBTHAUMO2_560020 | IBTHAUMv1_23780003 | 100 | 120 | 121 | 144 | 2.04E-87 | 248 | 99.17 | 82.64 |
|  | IBTHAUMO2_1100079 | IBTHAUMv1_4230002 | 100 | 172 | 172 | 354 | 6.57E-127 | 358 | 100.00 | 48.31 |
|  | IBTHAUMO2_1110001 | IBTHAUMv1_14670003 | 100 | 147 | 147 | 339 | 1.13E-106 | 305 | 100.00 | 43.07 |
|  | IBTHAUMO2_20011 | IBTHAUMv1_18170003 | 100 | 119 | 119 | 454 | 1.75E-82 | 246 | 100.00 | 25.99 |
| Integrases | IBTHAUMO2_240171 | IBTHAUMv1_2410001 | 99.74 | 385 | 557 | 387 | 0 | 801 | 69.12 | 99.22 |
|  | IBTHAUMO2_450024 | IBTHAUMv1_16200001 | 100 | 454 | 454 | 472 | 0 | 939 | 100.00 | 95.97 |
|  | IBTHAUMO2_730023 | IBTHAUMv1_1500005 | 98.063 | 413 | 448 | 424 | 0 | 840 | 92.19 | 96.93 |
|  | IBTHAUMO2_880010 | IBTHAUMv1_16080009 | 99.787 | 469 | 469 | 469 | 0 | 967 | 100.00 | 99.79 |
|  | IBTHAUMO2_780006 | IBTHAUMv1_11050003 | 99.674 | 307 | 307 | 343 | 0 | 628 | 100.00 | 89.21 |
| Restriction-modification | IBTHAUMO2_1050026 | IBTHAUMv1_1120001 | 25.738 | 237 | 927 | 640 | 2.47E-06 | 48.5 | 25.57 | 31.41 |
| systems | IBTHAUMO2_1130086 | IBTHAUMv1_13350012 | 25.895 | 475 | 612 | 1038 | 8.26E-32 | 129 | 77.61 | 45.47 |
|  | IBTHAUMO2_260066 | IBTHAUMv1_14890001 | 99.05 | 421 | 1333 | 586 | 0 | 863 | 31.58 | 71.67 |
|  | IBTHAUMO2_700006 | IBTHAUMv1_4230001 | 100 | 887 | 953 | 889 | 0 | 1845 | 93.07 | 99.66 |
|  | IBTHAUMO2_880011 | IBTHAUMv1_13350012 | 20.659 | 334 | 908 | 1038 | 2.49E-06 | 48.9 | 36.78 | 31.21 |
|  | IBTHAUMO2_890002 | IBTHAUMv1_13350012 | 99.711 | 1038 | 1037 | 1038 | 0 | 2118 | 100.10 | 99.90 |
|  | IBTHAUMO2_240074 | IBTHAUMv1_15140004 | 99.184 | 490 | 490 | 490 | 0 | 975 | 100.00 | 99.80 |
|  | IBTHAUMO2_240151 | IBTHAUMv1_16180005 | 100 | 574 | 574 | 582 | 0 | 1178 | 100.00 | 98.45 |
|  | IBTHAUMO2_280001 | IBTHAUMv1_11110026 | 98.119 | 638 | 675 | 729 | 0 | 1280 | 94.52 | 87.38 |
|  | IBTHAUMO2_380002 | IBTHAUMv1_11240027 | 99.42 | 862 | 862 | 862 | 0 | 1708 | 100.00 | 99.88 |
|  | IBTHAUMO2_730017 | IBTHAUMv1_3930004 | 99.719 | 711 | 711 | 711 | 0 | 1407 | 100.00 | 99.86 |
|  | IBTHAUMO2_800038 | IBTHAUMv1_3940004 | 100 | 648 | 648 | 648 | 0 | 1320 | 100.00 | 99.85 |
|  | IBTHAUMO2_260067 | IBTHAUMv1_980001 | 97.107 | 242 | 271 | 273 | 1.02E-174 | 480 | 89.30 | 88.28 |
|  | IBTHAUMO2_590086 | IBTHAUMv1_1620001 | 35 | 60 | 854 | 856 | 3.10E-01 | 32 | 7.03 | 6.89 |
|  | IBTHAUMO2_790008 | IBTHAUMv1_19180002 | 90.868 | 438 | 439 | 442 | 0 | 823 | 99.77 | 98.87 |
|  | IBTHAUMO2_390013 | IBTHAUMv1_14530025 | 99.27 | 274 | 290 | 283 | 0 | 559 | 94.48 | 96.47 |
|  | IBTHAUMO2_590085 | IBTHAUMv1_9460007 | 29.957 | 464 | 633 | 608 | 3.18E-52 | 187 | 73.30 | 63.65 |
|  | IBTHAUMO2_590087 | IBTHAUMv1_9460007 | 35.641 | 390 | 594 | 608 | 7.75E-62 | 213 | 65.66 | 59.38 |
|  | IBTHAUMO2_740003 | IBTHAUMv1_70001 | 66 | 450 | 446 | 477 | 0 | 639 | 100.90 | 94.13 |
|  | IBTHAUMO2_320013 | IBTHAUMv1_3540002 | 100 | 281 | 281 | 281 | 0 | 561 | 100.00 | 99.64 |
|  | IBTHAUMO2_390007 | IBTHAUMv1_14530029 | 98.233 | 283 | 299 | 287 | 0 | 558 | 94.65 | 98.26 |
|  | IBTHAUMO2_770023 | IBTHAUMv1_19010011 | 100 | 269 | 269 | 269 | 0 | 558 | 100.00 | 99.63 |
|  | IBTHAUMO2_240101 | IBTHAUMv1_10080009 | 99.569 | 464 | 510 | 467 | 0 | 925 | 90.98 | 99.14 |
|  | IBTHAUMO2_590091 | IBTHAUMv1_7470001 | 93.216 | 398 | 462 | 399 | 0 | 763 | 86.15 | 99.50 |
|  | IBTHAUMO2_690028 | IBTHAUMv1_3820007 | 30.699 | 329 | 377 | 379 | 8.32E-33 | 124 | 87.27 | 79.68 |
|  | IBTHAUMO2_810008 | IBTHAUMv1_7470001 | 45.013 | 391 | 474 | 399 | 1.25E-98 | 300 | 82.49 | 94.99 |
| Toxin: antitoxin systems | IBTHAUMO2_1130060 | IBTHAUMv1_13940004 | 100 | 83 | 83 | 83 | 2.73E-58 | 170 | 100.00 | 98.80 |
|  | IBTHAUMO2_990027 | IBTHAUMv1_13940004 | 34.667 | 75 | 86 | 83 | 2.83E-11 | 52 | 87.21 | 87.95 |
|  | IBTHAUMO2_570004 | IBTHAUMv1_840006 | 98.214 | 112 | 112 | 112 | 5.96E-77 | 220 | 100.00 | 99.11 |
|  | IBTHAUMO2_590016 | IBTHAUMv1_23220003 | 98.942 | 378 | 378 | 378 | 0 | 740 | 100.00 | 99.74 |
|  | IBTHAUMO2_330015 | IBTHAUMv1_1010002 | 99.091 | 330 | 377 | 330 | 0 | 655 | 87.53 | 99.70 |
|  | IBTHAUMO2_520002 | IBTHAUMv1_4560007 | 100 | 376 | 377 | 376 | 0 | 768 | 99.73 | 99.73 |
|  | IBTHAUMO2_210021 | IBTHAUMv1_6980001 | 100 | 294 | 402 | 294 | 0 | 598 | 73.13 | 99.66 |
|  | IBTHAUMO2_440006 | IBTHAUMv1_15080002 | 99.785 | 465 | 465 | 465 | 0 | 900 | 100.00 | 99.78 |
|  | IBTHAUMO2_870005 | IBTHAUMv1_5820001 | 100 | 214 | 447 | 216 | 1.21E-150 | 424 | 47.87 | 98.61 |
|  | IBTHAUMO2_940001 | IBTHAUMv1_4550011 | 100 | 418 | 419 | 465 | 0 | 835 | 99.76 | 89.68 |

| **Table S3**. Expressed proteins assigned to the *I. basta* thaumarchaeote MAG ordered by NSAF value. Proteins that are encoded exclusively by *Ca*. N. ianthellae among the AOA are labeled in orange. Proteins encoded by all AOA are labeled in blue. | | | | | | | | | | | |
| --- | --- | --- | --- | --- | --- | --- | --- | --- | --- | --- | --- |
| **Accession** | **Gene** | **Description** | **AA length** | **NSAF** | **OG Family** | **Presence in 454 Thaumarchaeal bin** | | | | **Distribution of OGs**** | **Notes** |
|  |  |  |  |  |  | **Accession** | **maxLrap*** | **minLrap*** | **% BLASTp hit** |  |  |
| IBTHAUMO2_1100065 |  | 4Fe-4S ferredoxin | 100 | **9.23%** | OG0000047 | IBTHAUMv1_12290026 | 1 | 1 | 100 | Core |  |
| IBTHAUMO2_240128 | *tuf* | Elongation factor 1-alpha | 432 | **5.90%** | OG0000743 | IBTHAUMv1_310002 | 1 | 1 | 100 | Core |  |
| IBTHAUMO2_720032 | *nirK* | putative nitrite reductase, copper-dependent | 468 | **3.95%** | OG0000247 | IBTHAUMv1_10010001 | 0.46795 | 0.99095 | 96.35 | not in *Ca*. N. islandicus and *Ca.* C. symbisoum |  |
| IBTHAUMO2_320009 | *amoB* | putative archaeal ammonia monooxygenase subunit B | 189 | **3.49%** | OG0000306 | IBTHAUMv1_240002 | 1 | 1 | 100 | Core |  |
| IBTHAUMO2_950017 |  | conserved protein of unknown function | 355 | **3.34%** | OG0000097 | IBTHAUMv1_4200003 | 0.58028 | 0.78626 | 89.32 |  |  |
| IBTHAUMO2_890024 | *ths* | Thermosome subunit | 546 | **3.22%** | OG0000022 | IBTHAUMv1_7040001 | 1 | 1 | 99.82 | Core |  |
| IBTHAUMO2_240148 |  | Zn-dependent oxidoreductase | 356 | **3.09%** | OG0000035 | IBTHAUMv1_16180002 | 1 | 1 | 100 | Core |  |
| IBTHAUMO2_1130066 |  | conserved protein of unknown function | 86 | **3.07%** | OG0001007 | IBTHAUMv1_620009 | 1 | 1 | 100 |  |  |
| IBTHAUMO2_1100022 | *ths* | Thermosome subunit | 566 | **3.03%** | OG0000022 | IBTHAUMv1_4580008 | 1 | 1 | 99.82 | Core |  |
| IBTHAUMO2_510038 | *rrp41* | Exosome complex component Rrp41 | 243 | **2.53%** | OG0000833 | IBTHAUMv1_1960004 | 1 | 1 | 100 | Core |  |
| IBTHAUMO2_250013 | *trxA* | Thioredoxin 1 | 108 | **2.44%** | OG0000373 | IBTHAUMv1_11050023 | 1 | 1 | 100 | Core |  |
| IBTHAUMO2_170030 | *atpA* | V-type ATP synthase alpha chain | 589 | **2.39%** | OG0000512 | IBTHAUMv1_3990024 | 1 | 1 | 100 | Core |  |
| IBTHAUMO2_320010 | *amoC* | Ammonia monooxygenase/methane monooxygenase, subunit C | 187 | **2.35%** | OG0000065 | IBTHAUMv1_4820001 | 0.92118 | 1 | 100 | Core |  |
| IBTHAUMO2_1070007 |  | Band 7 protein | 285 | **2.31%** | OG0001373 | IBTHAUMv1_16780016 | 0.8807 | 1 | 100 |  |  |
| IBTHAUMO2_1110035 | *psmA* | Proteasome subunit alpha 2 | 240 | **1.83%** | OG0000072 | IBTHAUMv1_1190006 | 1 | 1 | 100 | Core |  |
| IBTHAUMO2_270002 |  | protein of unknown function | 986 | **1.74%** | OG0001421 | IBTHAUMv1_16090001 | 0.91481 | 1 | 100 | All 3 sponge symbionts, 2 *Ca*. Nitrosotaleales, 1 *Ca*. N. brevis | s-layer protein family |
| IBTHAUMO2_780001 |  | exported protein of unknown function | 511 | **1.63%** | OG0001421 | IBTHAUMv1_2030001 | 0.48711 | 0.99804 | 100 | All 3 sponge symbionts, 2 *Ca*. Nitrosotaleales, 1 *Ca*. N. brevis | s-layer protein family |
| IBTHAUMO2_450045 | *sufC* | FeS assembly ATPase SufC | 256 | **1.55%** | OG0000878 | IBTHAUMv1_9550001 | 1 | 1 | 100 | Core |  |
| IBTHAUMO2_1070037 |  | 4Fe-4S ferredoxin | 181 | **1.46%** | OG0000029 | IBTHAUMv1_3930011 | 1 | 1 | 100 | Core |  |
| IBTHAUMO2_1130095 | *livK* | ABC-type branched-chain amino acid transport system, periplasmic component (modular protein) | 526 | **1.42%** | OG0001679 | IBTHAUMv1_16960002 | 1 | 1.03137 | 93.73 | All 3 sponge symbionts, *N. maritimus* and both Nitrosocosmicus spp. |  |
| IBTHAUMO2_660006 |  | exported protein of unknown function | 510 | **1.38%** | OG0001421 | IBTHAUMv1_2030001 | 0.48902 | 1.00392 | 82.62 | All 3 sponge symbionts, 2 *Ca*. Nitrosotaleales, 1 *Ca*. N. brevis | s-layer protein family |
| IBTHAUMO2_1030029 |  | putative archaeal aspartate aminotransferase | 383 | **1.38%** | OG0000543 | IBTHAUMv1_3950021 | 1 | 1 | 100 | Core |  |
| IBTHAUMO2_240080 |  | exported protein of unknown function | 2072 | **1.32%** | OG0001466 | IBTHAUMv1_11360001 | 1 | 1 | 98.65 | *Ca.* N. ianthellae unique | DUF5011 domain-containing |
| IBTHAUMO2_990028 | *psmA* | Proteasome subunit alpha | 244 | **1.26%** | OG0000072 | IBTHAUMv1_15240015 | 1 | 1 | 100 | Core |  |
| IBTHAUMO2_60001 |  | exported protein of unknown function | 1110 | **1.23%** | OG0001421 | IBTHAUMv1_15240010 | 0.71754 | 1.05045 | 62.78 | All 3 sponge symbionts, 2 *Ca*. Nitrosotaleales, 1 *Ca*. N. brevis | s-layer protein family |
| IBTHAUMO2_320005 | *rps15* | 30S ribosomal protein S15 | 149 | **1.18%** | OG0000795 | IBTHAUMv1_240006 | 1 | 1 | 99.33 | Core |  |
| IBTHAUMO2_170031 | *atpB* | V-type ATP synthase beta chain | 456 | **1.16%** | OG0000511 | IBTHAUMv1_3990023 | 1 | 1 | 100 | Core |  |
| IBTHAUMO2_980016 | *rpoA* | DNA-directed RNA polymerase subunit A'' | 1263 | **1.11%** | OG0000600 | IBTHAUMv1_12910016 | 1 | 1 | 100 | Core |  |
| IBTHAUMO2_450036 |  | protein of unknown function | 82 | **1.07%** | OG0002396 | IBTHAUMv1_15190002 | 1 | 1 | 90.24 | Only shared b/n *Ca*. N. ianthellae, *N. maritimus*, and *Ca*. N. gargensis |  |
| IBTHAUMO2_950026 | *ftnB* | putative ferritin-2 | 168 | **1.05%** | OG0001406 | IBTHAUMv1_3240002 | 1 | 1 | 99.4 | Sporadic distribution - seems to be concentrated in the Nitrosopumiliacae |  |
| IBTHAUMO2_530013 | *albA* | DNA/RNA-binding protein Alba (modular protein) | 170 | **1.03%** | OG0000027 | IBTHAUMv1_3270004 | 1 | 1 | 100 | Core |  |
| IBTHAUMO2_980006 | *mdh* | Malate dehydrogenase | 302 | **1.02%** | OG0000569 | IBTHAUMv1_4300002 | 0.91515 | 1 | 98.34 | Core |  |
| IBTHAUMO2_240105 |  | conserved exported protein of unknown function | 451 | **0.97%** | OG0000059 | IBTHAUMv1_1870005 | 0.55654 | 1 | 100 |  |  |
| IBTHAUMO2_770040 |  | Cyclase/dehydrase | 198 | **0.89%** | OG0000265 | IBTHAUMv1_20630002 | 1 | 1 | 100 | Core |  |
| IBTHAUMO2_980019 |  | Ribosomal protein L7Ae/L30e/S12e/Gadd45 | 104 | **0.85%** | OG0000601 | IBTHAUMv1_12970010 | 1 | 1 | 100 | Core |  |
| IBTHAUMO2_1110009 |  | conserved exported protein of unknown function | 313 | **0.84%** | OG0000016 | IBTHAUMv1_920001 | 0.85942 | 1 | 99.26 | Core |  |
| IBTHAUMO2_1110029 | *erpA* | Iron-sulfur cluster insertion protein ErpA 1 | 116 | **0.76%** | OG0000603 | IBTHAUMv1_18460002 | 1 | 1 | 100 | Core |  |
| IBTHAUMO2_1110033 | *gdhA* | Glutamate dehydrogenase | 424 | **0.73%** | OG0000604 | IBTHAUMv1_18460006 | 0.80189 | 1 | 100 | Core |  |
| IBTHAUMO2_990049 | *rpl6* | 50S ribosomal protein L6 | 182 | **0.72%** | OG0000737 | IBTHAUMv1_4350015 | 1 | 1 | 99.45 | Core |  |
| IBTHAUMO2_720011 |  | exported protein of unknown function | 1424 | **0.71%** | OG0001466 | IBTHAUMv1_4130010 | 1 | 1 | 99.16 | *Ca.* N. ianthellae unique |  |
| IBTHAUMO2_470003 | *tbp* | TATA-box-binding protein | 187 | **0.71%** | OG0000031 | IBTHAUMv1_12200005 | 1 | 1 | 100 | Core |  |
| IBTHAUMO2_240046 |  | conserved protein of unknown function | 130 | **0.68%** | OG0000007 | IBTHAUMv1_2100004 | 1 | 1 | 100 | only missing in *Ca.*N. islandicus |  |
| IBTHAUMO2_980022 | *rps7* | 30S ribosomal protein S7 | 199 | **0.66%** | OG0000837 | IBTHAUMv1_13050003 | 1 | 1 | 100 | Core |  |
| IBTHAUMO2_220008 | *sodA* | Superoxide dismutase [Mn] | 205 | **0.64%** | OG0000084 | IBTHAUMv1_13830025 | 1 | 1 | 100 | Core |  |
| IBTHAUMO2_260024 |  | Universal stress protein (UspA domain-containing protein) | 141 | **0.62%** | OG0000011 | IBTHAUMv1_1640004 | 1 | 1 | 100 | only missing in *Ca.*N. islandicus | important for oxidative and acid stress |
| IBTHAUMO2_260038 |  | Cupin 2 conserved barrel domain protein | 142 | **0.62%** | OG0001023 | IBTHAUMv1_1140005 | 1 | 1 | 99.3 | only missing in *Ca.*N. islandicus |  |
| IBTHAUMO2_340008 | *dnaK* | Chaperone protein DnaK | 503 | **0.61%** | OG0000335 | IBTHAUMv1_11380008 | 0.75262 | 0.99801 | 100 | Core |  |
| IBTHAUMO2_210018 | *cofD* | LPPG:FO 2-phospho-L-lactate transferase | 306 | **0.57%** | OG0000676 | IBTHAUMv1_4620004 | 1 | 1 | 100 | Core | F420 biosynthesis |
| IBTHAUMO2_1110028 | *dnaG* | DNA primase DnaG | 386 | **0.57%** | OG0000693 | IBTHAUMv1_2640008 | 0.80829 | 1 | 99.68 | Core |  |
| IBTHAUMO2_1030019 | *ppi* | putative peptidyl-prolyl cis-trans isomerase | 158 | **0.56%** | OG0000324 | IBTHAUMv1_12830004 | 1 | 1 | 100 | only missing in *Ca.*N. islandicus |  |
| IBTHAUMO2_210017 |  | conserved exported protein of unknown function | 448 | **0.49%** | OG0000096 | IBTHAUMv1_1340002 | 1 | 1 | 100 | Core |  |
| IBTHAUMO2_320015 |  | Alkyl hydroperoxide reductase | 182 | **0.48%** | OG0000941 | IBTHAUMv1_2320004 | 1 | 1 | 99.45 | only missing in *Ca.*N. salalaria |  |
| IBTHAUMO2_240129 | *fbp* | bifunctional fructose-1,6-bisphosphatase | 376 | **0.47%** | OG0000417 | IBTHAUMv1_310001 | 0.97606 | 1 | 99.46 | Core |  |
| IBTHAUMO2_1130058 | *pepA* | putative leucyl aminopeptidase | 477 | **0.46%** | OG0001191 | IBTHAUMv1_13940006 | 1 | 1 | 99.79 | missing in *Ca.* N. islandicus and all *Ca.* Nitrosotaleales |  |
| IBTHAUMO2_720005 | *rpoE* | DNA-directed RNA polymerase subunit E' | 193 | **0.46%** | OG0000408 | IBTHAUMv1_4130016 | 1 | 1 | 100 | Core |  |
| IBTHAUMO2_1130033 | *accC/ pccC* | acetyl-CoA/propionyl-CoA carboxylase, biotin carboxylase subunit | 485 | **0.45%** | OG0000362 | IBTHAUMv1_13940030 | 1 | 1 | 100 | Core |  |
| IBTHAUMO2_720031 |  | NAD-binding D-isomer specific 2-hydroxyacid dehydrogenase | 310 | **0.43%** | OG0000385 | IBTHAUMv1_4660002 | 1 | 1 | 97.42 | Core |  |
| IBTHAUMO2_990024 | *fusA* | Elongation factor 2 | 730 | **0.42%** | OG0000645 | IBTHAUMv1_16760020 | 1 | 1 | 99.73 | Core |  |
| IBTHAUMO2_720043 |  | exported protein of unknown function | 1462 | **0.42%** | OG0001466 | IBTHAUMv1_5230001 | 0.70588 | 0.99807 | 95.35 | *Ca.* N. ianthellae unique |  |
| IBTHAUMO2_1100034 |  | Peptidyl-prolyl cis-trans isomerase | 533 | **0.41%** | OG0000003 | IBTHAUMv1_17580009 | 1 | 1 | 99.25 | only missing in both Nitrosocosmicus spp. |  |
| IBTHAUMO2_40002 |  | conserved protein of unknown function | 976 | **0.41%** | OG0000006 | IBTHAUMv1_3870008 | 0.61226 | 1.03381 | 75.22 | only missing in both Nitrosocosmicus spp. | s-layer protein family |
| IBTHAUMO2_240132 |  | conserved protein of unknown function | 217 | **0.41%** | OG0000436 | IBTHAUMv1_5220002 | 1 | 1 | 100 | Core | contains PF01865 domain - putative phosphate transport regulator |
| IBTHAUMO2_1080008 |  | exported protein of unknown function | 557 | **0.39%** | OG0001421 | IBTHAUMv1_15240001 | 0.33504 | 0.93896 | 62.33 | All 3 sponge symbionts, 2 *Ca*. Nitrosotaleales, 1 *Ca*. N. brevis | s-layer protein family |
| IBTHAUMO2_240136 |  | Short-chain dehydrogenase/reductase SDR | 574 | **0.38%** | OG0000810 | IBTHAUMv1_1110007 | 1 | 1 | 99.48 | Core |  |
| IBTHAUMO2_980023 |  | membrane protein of unknown function | 586 | **0.38%** | OG0011727 | IBTHAUMv1_12910009 | 1 | 1 | 99.83 | singleton |  |
| IBTHAUMO2_1110030 |  | 3-hydroxypropionyl-CoA dehydratase/Crotonyl-CoA hydratase [(S)-3-hydroxybutyryl-CoA forming] | 252 | **0.35%** | OG0000227 | IBTHAUMv1_18460003 | 1 | 1 | 100 | Core |  |
| IBTHAUMO2_510039 | *rrp42* | Exosome complex component Rrp42 | 270 | **0.33%** | OG0000834 | IBTHAUMv1_1960005 | 1 | 1 | 100 | Core |  |
| IBTHAUMO2_1100023 | *glyA* | Serine hydroxymethyltransferase | 448 | **0.29%** | OG0000541 | IBTHAUMv1_4580009 | 0.97545 | 1 | 100 | Core |  |
| IBTHAUMO2_940015 | *sucD* | succinyl-CoA synthetase, NAD(P)-binding, alpha subunit | 302 | **0.29%** | OG0000763 | IBTHAUMv1_330006 | 1 | 1 | 99.34 | Core |  |
| IBTHAUMO2_590109 | *sufD* | FeS assembly protein | 461 | **0.29%** | OG0000995 | IBTHAUMv1_4640007 | 1 | 1 | 100 | only missing in *Ca.*N. salalaria |  |
| IBTHAUMO2_800011 |  | putative aldolase | 314 | **0.28%** | OG0000682 | IBTHAUMv1_14600002 | 1 | 1.0129 | 98.73 | Core | putatively functional in E.C. 4.1.2.13 |
| IBTHAUMO2_590122 | *hemB* | Delta-aminolevulinic acid dehydratase | 321 | **0.27%** | OG0000183 | IBTHAUMv1_830005 | 0.85981 | 1 | 100 | Core |  |
| IBTHAUMO2_260012 | *trxB* | Thioredoxin reductase | 327 | **0.27%** | OG0000820 | IBTHAUMv1_4840006 | 0.93162 | 1 | 99.69 | Core |  |
| IBTHAUMO2_1080004 |  | conserved exported protein of unknown function | 508 | **0.26%** | OG0000016 | IBTHAUMv1_16190003 | 1 | 1 | 99.41 | Core |  |
| IBTHAUMO2_170024 |  | H(+)-transporting two-sector ATPase | 340 | **0.26%** | OG0000137 | IBTHAUMv1_2710003 | 1 | 1 | 100 | Core |  |
| IBTHAUMO2_950011 | *aroB* | 3-dehydroquinate synthase | 341 | **0.26%** | OG0000823 | IBTHAUMv1_560007 | 1 | 1 | 96.19 | Core | tyrosine, tryptophan, phe synthesis |
| IBTHAUMO2_170028 | *atpI* | V-type ATP synthase subunit I | 694 | **0.25%** | OG0001210 | IBTHAUMv1_15570002 | 0.62104 | 1 | 100 | Core |  |
| IBTHAUMO2_1110031 |  | 3-Hydroxypropionyl-CoA synthetase | 704 | **0.25%** | OG0000680 | IBTHAUMv1_18460004 | 1 | 1 | 100 | Core |  |
| IBTHAUMO2_770013 |  | AAA family ATPase, CDC48 subfamily | 728 | **0.24%** | OG0000098 | IBTHAUMv1_11430014 | 1 | 1 | 100 | Core |  |
| IBTHAUMO2_300003 |  | Redoxin domain-containing protein | 371 | **0.24%** | OG0000167 | IBTHAUMv1_13230016 | 1 | 1 | 99.73 | only missing in *Ca.*N. islandicus |  |
| IBTHAUMO2_470029 |  | Beta-lactamase domain-containing protein | 421 | **0.21%** | OG0000769 | IBTHAUMv1_1130003 | 1 | 1 | 99.76 | Core |  |
| IBTHAUMO2_260042 | *hemL* | Glutamate-1-semialdehyde 2,1-aminomutase 2 | 441 | **0.20%** | OG0000389 | IBTHAUMv1_13740001 | 0.92517 | 0.99756 | 99.75 | Core | F430/tetrapyrrole/B12 biosynthesis |
| IBTHAUMO2_980015 | *rpoB* | DNA-directed RNA polymerase subunit B | 1115 | **0.20%** | OG0000599 | IBTHAUMv1_1480002 | 0.71031 | 0.99874 | 99.87 | Core |  |
| IBTHAUMO2_370006 | *cysM* | Cysteine synthase | 492 | **0.18%** | OG0001268 | IBTHAUMv1_12200010 | 1 | 1 | 98.78 | missing in *Ca.* N. islandicus, both Nitrosocosmicus spp., all *Ca*. Nitrosotenuaceae |  |
| IBTHAUMO2_690011 |  | conserved exported protein of unknown function | 492 | **0.18%** | OG0000003 | IBTHAUMv1_11050007 | 1 | 1 | 99.59 | not in both Nitrosocosmicus and many AOA have multiple copies | SSF51004: Cytochrome cd1-nitrite reductase-like, haem d1 domain superfamily |
| IBTHAUMO2_320044 | *aco* | Aconitate hydratase | 745 | **0.18%** | OG0000268 | IBTHAUMv1_4090010 | 1 | 1 | 99.46 | Core |  |
| IBTHAUMO2_370005 |  | von Willebrand factor type A | 498 | **0.18%** | OG0000623 | IBTHAUMv1_12200011 | 0.58635 | 1 | 99.66 | Core | COG4548: P, Nitric oxide reductase activation protein |
| IBTHAUMO2_330024 | *pstS* | ABC-type phosphate transport system periplasmic component (PstS) | 508 | **0.17%** | OG0000113 | IBTHAUMv1_17840008 | 1 | 1 | 99.41 | missing in *Ca.* N. salaria, *Ca.*N. koreensis AR2, *Ca*. N. sediminis, and *Ca*. N. catalina |  |
| IBTHAUMO2_170026 | *amt2* | Ammonium transporter | 520 | **0.17%** | OG0000221 | IBTHAUMv1_15380013 | 0.96923 | 1 | 100 | missing in both Nitrosocosmicus spp. |  |
| IBTHAUMO2_620022 |  | protein of unknown function | 1844 | **0.17%** | OG0001466 | IBTHAUMv1_12370012 | 0.85016 | 1 | 98.86 | *Ca.* N. ianthellae unique | DUF5011 domain-containing |
| IBTHAUMO2_960004 | *ths* | Thermosome subunit | 560 | **0.16%** | OG0002000 | IBTHAUMv1_4580008 | 0.89223 | 0.90179 | 42.57 |  |  |
| IBTHAUMO2_530015 | *sdhA* | Succinate dehydrogenase or fumarate reductase, flavoprotein subunit | 570 | **0.15%** | OG0000437 | IBTHAUMv1_4000002 | 0.92632 | 1 | 98.48 | Core |  |
| IBTHAUMO2_590087 |  | Type III restriction-modification system methyltransferase | 593 | **0.15%** | OG0001596 | IBTHAUMv1_9460007 | 0.95058 | 0.97302 | 31.37 |  |  |
| IBTHAUMO2_240089 | *ppdK* | Pyruvate, phosphate dikinase | 884 | **0.10%** | OG0001061 | IBTHAUMv1_4310008 | 1 | 1 | 100 |  |  |
| IBTHAUMO2_700001 |  | protein of unknown function | 3094 | **0.10%** | OG0001466 | IBTHAUMv1_20360004 | 0.50517 | 0.96125 | 99.42 | *Ca.* N. ianthellae unique | DUF5011 domain-containing |
| IBTHAUMO2_880008 |  | exported protein of unknown function | 2027 | **0.04%** | OG0000017 | IBTHAUMv1_19190004 | 0.32215 | 0.88243 | 46.86 |  | putative M72 metalloendopeptidase |
| *These values are ratios of alignment lengths computed for each comparison using the BLAST software : | | | | | | |  |  |  |  |  |
| minLrap = Lmatch/min(Lprot1, Lprot2) | | |  |  |  |  |  |  |  |  |  |
| maxLrap = Lmatch/max(Lprot1, Lprot2) | | |  |  |  |  |  |  |  |  |  |
| where Lmatch = length of the match, Lprot1 = length of protein 1, Lprot2 = length of protein 2 | | | | | | |  |  |  |  |  |
| **if minLrap=1 and maxLrap=1** => the 2 proteins both align on their whole length | | | | | |  |  |  |  |  |  |
| **if minLrap=1 and maxLrap<1** => one of the proteins is longer than the other, or the alignment is partial. | | | | | | |  |  |  |  |  |
|  |  |  |  |  |  |  |  |  |  |  |  |
| singletons refer to genes which did not have a hit above an expected threshold in OrthoFinder amongst all queried sequences | | | | | | | | |  |  |  |
|  |  |  |  |  |  |  |  |  |  |  |  |
| **If certain AOA do not have members in certain orthologous groups it does not mean that a more distant homologous gene is not present | | | | | | | | | | |  |

**Table S4.** Comparison of net nitrite formation and nitrification rates from our study with previously published sponge studies.

| **Sponge species** | **Net nitrification rates [µmol N (cm^-3^ or g^‑1^ wet wt.) day^-1^]** | **Net nitrite formation rates [µmol N (cm^-3^ or g^‑1^ wet wt.) day^-1^]** | **Marine area, depth, and season** | **Experimental Setup** | **AOB/AOA Diversity** | **Reference** |
| --- | --- | --- | --- | --- | --- | --- |
| ***Anthosigmella varians***  ***Chondrilla nucula*** | 0.003 – 0.105 (g­^-1^*)  1.03 – 1.49 ◊ (g­^-1^*) | N.D. | Caribbean coral reef (6m) | 4h - 2.25 L batch incubations +/- light and 5 µM NH^­^_4_^+^ | N.D. | Corredor *et al*., 1988 |
| ***Chondrilla nucula***  ***Pseudaxinella zeai***  ***Oligoceras violacea***  ***Plakortis halichondroides*** | 0.864 – 6.36 (g­^-1^*)  0 – 2.47 (g­^-1^*)  0 – 1.37 (g­^-1^*)  0 – 0.768 (g­^-1^*) | 0.014 – 0.24 (g^-1^*)  0 – 0.048 (g^-1^*)  0.408 – 1.39 (g^-1^*)  0 – 0.192 (g^-1^*) | Caribbean coral reef (20-40m) and mangroves (1-3m); June-Sept. | 6 to 12h – 3 and 20L batch inc., +/- light | N.D. | Diaz & Ward, 1997 |
| ***Alpysina aerophoba***  ***Dysidea avara***  ***Chondrosia reniformis*** | 3.6 – 9.2 ◊ (g^-1^)  0  ~0.3 (g^-1^) | N.D. | Med. Sea, 2-20m; | 9 to 28h - 3L batch, +/- 100 or 200 µM NH_4_^+^ | β-AOB 16S – 9 OTUs   - 1 OTU | Bayer *et al*., 2007 |
| ***Alpysina aerophoba*** | 0.214 – 0.826 (g^-1^*) | N.D. | Med. Sea, 2-15m April - Sept. | 21 to 28h – 3L batch inc., +/- 100 or 200 µM NH_4_^+^  +/- nitrapyrin | A- and β-*amoA*/16S – 5, 7, 9 OTUs | Bayer *et al*., 2008 |
| ***Aplysina aerophoba***  ***Agelas oroides***  ***Dysidea avara***  ***Chondroisa reniformis***  ***Axinella polypoides***  ***Ircinia oros*** | 1.13 (g^-1^*)  0.875 (g­^-1^*)  N.S.  1.68 (g­^-1^*)  0.452 (g­^-1^*)  0.544 (g­^-1^*) | N.D. | Med. Sea, 10-20m; Sept. for all except for *A. oroides* (July) | 6h – 7L batch inc. | 6 A-*amoA* OTUs in 3 clust.  12 β-*amoA* OTUs in 3 clust.  83 γ-*amoA* clones | Jiménez & Ribes, 2007; Ribes *et al*., 2012 |
| ***Aplysina cauliformis***  ***Smenospongia aurea***  **7 other species: *A. archeri,* *A. lacunose, I. felix, I. strobilina, P. crassa, V. rigidia*, and *X. muta*** | 4.08 (g^-1^*)  4.32 (g­^-1^*)  + + + + + + + | N.D. | Florida Keys, ~4m | 6 to 8h – 2-4L batches; *in situ* sampling | N.D. | Southwell *et al*., 2008 |
| ***Chondrosia reniformis*,**  ***Dysidea avara*** | 0.176 (g^-1^)  0.294 (g^-1^) | 0.016 (g^-1^)  0.016 (g^-1^) | Med. Sea, 10-15m | 24h – 1L batches with 10 µM NH_4_^+^ | 1 A-*amoA* OTU | Schläppy *et al*., 2010 |
| ***Phakellia ventilabrum***  ***Antho dichotoma***  ***Geodia barretti*** | 0.14 – 2.26 (g^-1^○)  0.  0.679 (g^-1^○) | 0 – 0.192 (g^-1^○)  0.360 (g^-1^○)  0.180 (g^-1^○) | Norwegian coast (fjords), 200-300m; Mar.-Nov. | 24 to 48 h in 500 to 900 mL batches with 10-12 µM NH_4_^+^ | 3 A-*amoA* OTUs  3 A-*amoA* OTUs  1 A-*amoA* OTU | Radax *et al*., 2012; Hoffmann *et al*., 2009 |
| ***Ianthella basta*** | 1.67 – 11.3 (g^-1^)  1.54 – 13.3 ◊ (g^-1^) | 0.64 – 6.07 (g^-1^)  0.35 – 10.36 ◊ (g^-1^) | Coral Sea, Australia, 10 m; Sept. – Oct. | 7- day long 24 h batch incubations in 1.74 L  +/- 25, 100 µM NH_4_^+^  +/- PTIO | 1 A-*amoA* OTU | This study |

(***)** signifies that sponge dry wt. g^-1^ was converted by assuming dry weight = 10% of wet weight while a (○) signifies that sponge cm^-3^ was converted by assuming 1 cm^3^ sponge = 1.2 g wet weight sponge (Schläppy *et al*., 2010). A (◊) denotes that the rate is a potential rate (i.e. with added ammonium). A (+) indicates positive NO_x_^-^ production where quantity was not assessed. “A-, β- , and γ-*amoA*” refer to archaeal, betaproteobacterial and gammaproteobacterial *amoA* OTUs, respectively.


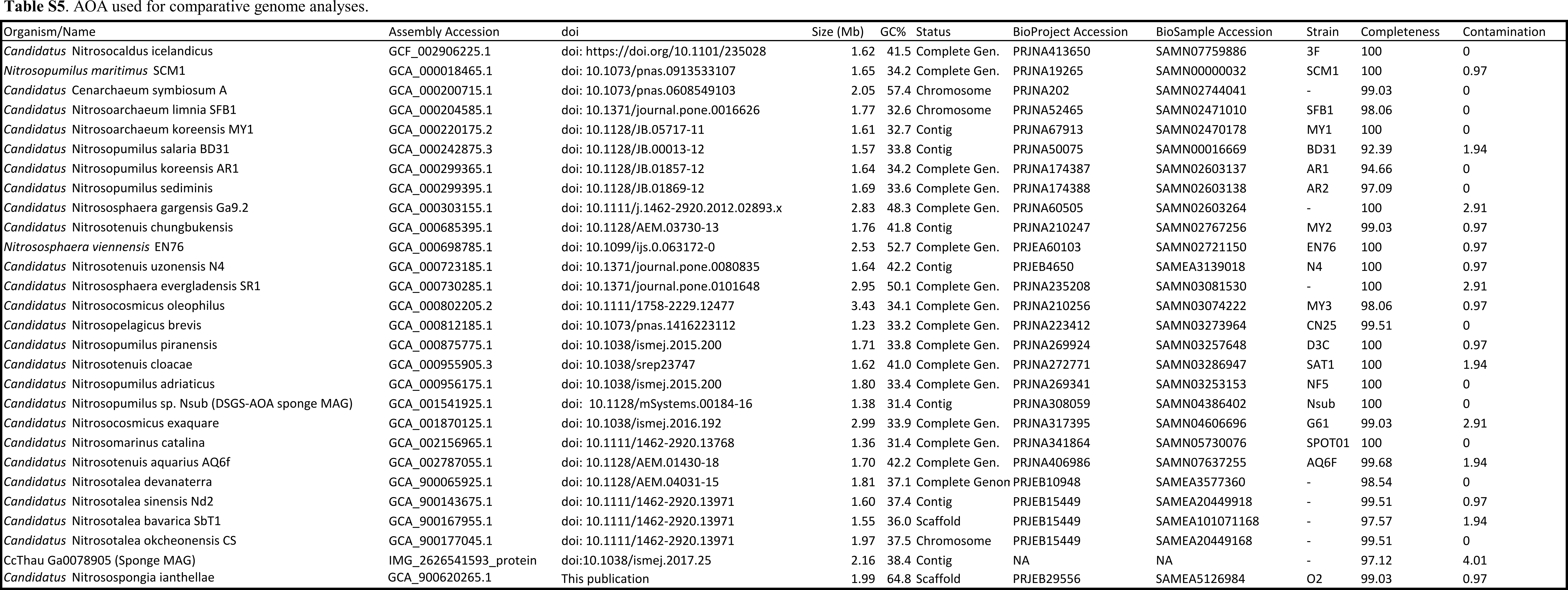


**References**

Alves, R.J.E., Minh, B.Q., Urich, T., von Haeseler, A., and Schleper, C. (2018) Unifying the global phylogeny and environmental distribution of ammonia-oxidising archaea based on *amoA* genes. *Nat Commun* **9**: 1517.

Bayer, K., Schmitt, S., and Hentschel, U. (2007) Microbial nitrification in Mediterranean sponges: possible involvement of ammonium‐oxidizing *Betaproteobacteria*. In *Porifera Research: Biodiversity, Innovation, Sustainability.*Custódio, M., Lôbo‐Hajdu, G., Hajdu, E., and Muricy, G. (eds). Série Livros. Rio de Janeiro, Brazil: Museu Nacional, pp. 165–171.

Bayer, K., Schmitt, S., and Hentschel, U. (2008) Physiology, phylogeny and *in situ* evidence for bacterial and archaeal nitrifiers in the marine sponge *Aplysina aerophoba*. *Environ Microbiol* **10**: 2942–2955.

Bayer, B., Vojvoda, J., Offre, P., Alves, R.J.E., Elisabeth, N.H., Garcia, J.A., *et al*. (2016) Physiological and genomic characterization of two novel marine thaumarchaeal strains indicates niche differentiation. *ISME J* **10**: 1051–1063.

Berger, S.A., Krompass, D., and Stamatakis, A. (2011) Performance, accuracy, and web Server for evolutionary placement of short sequence reads under maximum-likelihood. *Syst Biol* **60**: 291–302.

Blainey, P.C., Mosier, A.C., Potanina, A., Francis, C.A., and Quake, S.R. (2011) Genome of a low-salinity ammonia-oxidizing archaeon determined by single-cell and metagenomic analysis. *PLoS ONE* **6**: e16626.

Blatch, G.L. and Lässle, M. (1999) The tetratricopeptide repeat: a structural motif mediating protein-protein interactions. *Bioessays* **21**: 932–939.

Callebaut, I., Mornon, J.-P., Gilgès, D., and Vigon, I. (2000) HYR, an extracellular module involved in cellular adhesion and related to the immunoglobulin-like fold. *Protein Sci* **9**: 1382–1390.

Camacho, C., Coulouris, G., Avagyan, V., Ma, N., Papadopoulos, J., Bealer, K., and Madden, T.L. (2009) BLAST+: architecture and applications. *BMC Bioinformatics* **10**: 421.

Capella-Gutierrez, S., Silla-Martinez, J.M., and Gabaldon, T. (2009) trimAl: a tool for automated alignment trimming in large-scale phylogenetic analyses. *Bioinformatics* **25**: 1972–1973.

Cort, J.R., Yee, A., Edwards, A.M., Arrowsmith, C.H., and Kennedy, M.A. (2000) Structure-based functional classification of hypothetical protein MTH538 from *Methanobacterium thermoautotrophicum*. *J Mol Biol* **302**: 189–203.

Corredor, J.E., Wilkinson, C.R., Vicente, V.P., Morell, J.M., and Otero, E. (1988) Nitrate release by Caribbean reef sponges. *Limnol Oceanogr* **33**: 114–120.

Diaz, M. and Ward, B. (1997) Sponge-mediated nitrification in tropical benthic communities. *Mar Ecol Prog Ser* **156**: 97–107.

Doron, S., Melamed, S., Ofir, G., Leavitt, A., Lopatina, A., Keren, M., *et al*. (2018) Systematic discovery of antiphage defense systems in the microbial pangenome. *Science* **359**: eaar4120.

Edgar, R.C. (2004) MUSCLE: multiple sequence alignment with high accuracy and high throughput. *Nucleic Acids Res* **32**: 17921797.

Fan, L., Reynolds, D., Liu, M., Stark, M., Kjelleberg, S., Webster, N.S., and Thomas, T. (2012) Functional equivalence and evolutionary convergence in complex communities of microbial sponge symbionts. *Proc Natl Acad Sci USA* **109**: E1878–E1887.

Foerstner, K.U., von Mering, C., Hooper, S.D., and Bork, P. (2005) Environments shape the nucleotide composition of genomes. *EMBO Rep* **6**: 1208–1213.

Gauthier, M.E.A., Du Pasquier, L., and Degnan, B.M. (2010) The genome of the sponge Amphimedon queenslandica provides new perspectives into the origin of Toll-like and interleukin 1 receptor pathways: The origin of Toll-like and IL1 receptor pathways. *Evol Dev* **12**: 519–533.

Herbold, C.W., Lehtovirta-Morley, L.E., Jung, M.-Y., Jehmlich, N., Hausmann, B., Han, P., *et al*. (2017) Ammonia-oxidising archaea living at low pH: Insights from comparative genomics: Comparative genomics of ammonia-oxidising archaea. *Environ Microbiol* **19**: 4939–4952.

Hoffmann, F., Radax, R., Woebken, D., Holtappels, M., Lavik, G., Rapp, H.T., *et al*. (2009) Complex nitrogen cycling in the sponge *Geodia barretti*. *Environ Microbiol* **11**: 2228–2243.

Horn, H., Slaby, B.M., Jahn, M.T., Bayer, K., Moitinho-Silva, L., Förster, F., *et al*. (2016) An enrichment of CRISPR and other defense-related features in marine sponge-associated microbial metagenomes. *Front Microbiol* **7**: 1751.

Huerta-Cepas, J., Szklarczyk, D., Forslund, K., Cook, H., Heller, D., Walter, M.C., *et al*. (2016) eggNOG 4.5: a hierarchical orthology framework with improved functional annotations for eukaryotic, prokaryotic and viral sequences. *Nucleic Acids Res* **44**: D286–D293.

Jernigan, K.K. and Bordenstein, S.R. (2015) Tandem-repeat protein domains across the tree of life. *PeerJ* **3**: e732.

Jiménez, E. and Ribes, M. (2007) Sponges as a source of dissolved inorganic nitrogen: Nitrification mediated by temperate sponges. *Limnol Oceanogr* **52**: 948–958.

Jung, M.-Y., Park, S.-J., Kim, S.-J., Kim, J.-G., Sinninghe Damsté, J.S., Jeon, C.O., and Rhee, S.-K. (2014) A mesophilic, autotrophic, ammonia-oxidizing archaeon of thaumarchaeal group I.1a cultivated from a deep oligotrophic soil horizon. *Appl Environ Microbiol* **80**: 3645–3655.

Kalyaanamoorthy, S., Minh, B.Q., Wong, T.K.F., von Haeseler, A., and Jermiin, L.S. (2017) ModelFinder: fast model selection for accurate phylogenetic estimates. *Nat Methods* **14**: 587–589.

Karimi, E., Slaby, B.M., Soares, A.R., Blom, J., Hentschel, U., and Costa, R. (2018) Metagenomic binning reveals versatile nutrient cycling and distinct adaptive features in alphaproteobacterial symbionts of marine sponges. *FEMS Microbiol Ecol* **94**: 6.

Katoh, K. and Standley, D.M. (2013) MAFFT Multiple Sequence Alignment Software Version 7: Improvements in Performance and Usability. *Mol Biol Evol* **30**: 772–780.

Kerou, M., Offre, P., Valledor, L., Abby, S.S., Melcher, M., Nagler, M., *et al*. (2016) Proteomics and comparative genomics of *Nitrososphaera viennensis* reveal the core genome and adaptations of archaeal ammonia oxidizers. *Proc Natl Acad Sci USA*  **113**: E7937–E7946.

Könneke, M., Schubert, D.M., Brown, P.C., Hugler, M., Standfest, S., Schwander, T., *et al*. (2014) Ammonia-oxidizing archaea use the most energy-efficient aerobic pathway for CO2 fixation. *Proc Natl Acad Sci USA* **111**: 8239–8244.

Lagkouvardos, I., Joseph, D., Kapfhammer, M., Giritli, S., Horn, M., Haller, D., and Clavel, T. (2016) IMNGS: A comprehensive open resource of processed 16S rRNA microbial profiles for ecology and diversity studies. *Sci Rep-UK* **6**: 33721.

Lartillot, N. and Philippe, H. (2004) A Bayesian mixture model for across-site heterogeneities in the amino-acid replacement process. *Mol BiolEvol* **21**: 1095–1109.

Lassalle, F., Périan, S., Bataillon, T., Nesme, X., Duret, L., and Daubin, V. (2015) GC-Content evolution in bacterial genomes: the biased gene conversion hypothesis expands. *PLoS Genet* **11**: e1004941.

Laundon, D., Larson, B., McDonald, K., King, N., and Burkhardt, P. (2018) The architecture of cell differentiation in choanoflagellates and sponge choanocytes. *bioRxiv*.

Li, P.-N., Herrmann, J., Tolar, B.B., Poitevin, F., Ramdasi, R., Bargar, J.R., *et al*. (2018) Nutrient transport suggests an evolutionary basis for charged archaeal surface layer proteins. *ISME J* **12**: 2389–2402.

Mah, J.L., Christensen-Dalsgaard, K.K., and Leys, S.P. (2014) Choanoflagellate and choanocyte collar-flagellar systems and the assumption of homology. *Evol Dev* **16**: 25–37.

Maldonado, M. (2005) Choanoflagellates, choanocytes, and animal multicellularity. *Invertebr Biol* **123**: 1–22.

Manning, G., Young, S.L., Miller, W.T., and Zhai, Y. (2008) The protist, *Monosiga brevicollis*, has a tyrosine kinase signaling network more elaborate and diverse than found in any known metazoan. *Proc Natl Acad Sci USA* **105**: 9674–9679.

Moitinho-Silva, L., Díez-Vives, C., Batani, G., Esteves, A.I., Jahn, M.T., and Thomas, T. (2017) Integrated metabolism in sponge–microbe symbiosis revealed by genome-centered metatranscriptomics. *ISME J* **11**: 1651–1666.

Moitinho-Silva, L., Nielsen, S., Amir, A., Gonzalez, A., Ackermann, G.L., Cerrano, C., et al. (2017) The sponge microbiome project. *GigaScience* **6**: 1–7.

Mosier, A.C., Allen, E.E., Kim, M., Ferriera, S., and Francis, C.A. (2012) Genome sequence of ‘*Candidatus* Nitrosopumilus salaria’ BD31, an ammonia-oxidizing archaeon from the San Francisco Bay Estuary. *J Bacteriol* **194**: 2121–2122.

Nguyen, L.-T., Schmidt, H.A., von Haeseler, A., and Minh, B.Q. (2015) IQ-TREE: a fast and effective stochastic algorithm for estimating maximum-likelihood phylogenies. *Mol Biol Evol*  **32**: 268–274.

Otte, J., Mall, A., Schubert, D.M., Könneke, M., and Berg, I.A. (2015) Malonic semialdehyde reductase from the archaeon *Nitrosopumilus maritimus* is involved in the autotrophic 3-hydroxypropionate/4-hydroxybutyrate cycle. *Appl Environ Microbiol* **81**: 1700–1707.

Palatinszky, M., Herbold, C., Jehmlich, N., Pogoda, M., Han, P., von Bergen, M., *et al*. (2015) Cyanate as an energy source for nitrifiers. *Nature* **524**: 105–108.

Patterson, N.J., Günther, J., Gibson, A.J., Offord, V., Coffey, T.J., Splitter, G., *et al*. (2014) Two TIR-like domain containing proteins in a newly emerging zoonotic *Staphylococcus aureus* strain sequence type 398 are potential virulence factors by impacting on the host innate immune response. *Front Microbiol* **5**: 662.

Pruesse, E., Peplies, J., and Glöckner, F.O. (2012) SINA: Accurate high-throughput multiple sequence alignment of ribosomal RNA genes. *Bioinformatics* **28**: 1823–1829.

Qin, W., Meinhardt, K.A., Moffett, J.W., Devol, A.H., Virginia Armbrust, E., Ingalls, A.E., and Stahl, D.A. (2017) Influence of oxygen availability on the activities of ammonia-oxidizing archaea. *Envirol Microbiol Rep* **9**: 250–256.

Radax, R., Hoffmann, F., Rapp, H.T., Leininger, S., and Schleper, C. (2012) Ammonia-oxidizing archaea as main drivers of nitrification in cold-water sponges. *Environ Microbiol* **14**: 909–923.

Reichenberger, E.R., Rosen, G., Hershberg, U., and Hershberg, R. (2015) Prokaryotic nucleotide composition is shaped by both phylogeny and the environment. *Genome Biol Evol* **7**: 1380–1389.

Reynolds, D. and Thomas, T. (2016) Evolution and function of eukaryotic-like proteins from sponge symbionts. *Mol Ecol* **25**: 5242–5253.

Ribes, M., Jiménez, E., Yahel, G., López-Sendino, P., Diez, B., Massana, R., *et al*. (2012) Functional convergence of microbes associated with temperate marine sponges. *Environ Microbiol* **14**: 1224–1239.

Saier, M.H., Yen, M.R., Noto, K., Tamang, D.G., and Elkan, C. (2009) The Transporter Classification Database: recent advances. *Nucleic Acids Res* **37**: D274–D278.

Santoro, A.E., Dupont, C.L., Richter, R.A., Craig, M.T., Carini, P., McIlvin, M.R., *et al*. (2015) Genomic and proteomic characterization of “ *Candidatus* Nitrosopelagicus brevis”: An ammonia-oxidizing archaeon from the open ocean. *Proc Natl Acad Sci USA* **112**: 1173–1178.

Schläppy, M.-L., Schöttner, S.I., Lavik, G., Kuypers, M.M.M., de Beer, D., and Hoffmann, F. (2010) Evidence of nitrification and denitrification in high and low microbial abundance sponges. *Mar Biol* **157**: 593–602.

Shigeno-Nakazawa, Y., Kasai, T., Ki, S., Kostyanovskaya, E., Pawlak, J., Yamagishi, J., *et al*. (2016) A pre-metazoan origin of the CRK gene family and co-opted signaling network. *Sci Rep-UK* **6**: 3439.

Southwell, M.W., Popp, B.N., and Martens, C.S. (2008) Nitrification controls on fluxes and isotopic composition of nitrate from Florida Keys sponges. *Mar Chem* **108**: 96–108.

Spang, A., Poehlein, A., Offre, P., Zumbrägel, S., Haider, S., Rychlik, N., *et al*. (2012) The genome of the ammonia-oxidizing *Candidatus* Nitrososphaera gargensis: insights into metabolic versatility and environmental adaptations. *Environ Microbiol* **14**: 3122–3145.

Srivastava, M., Simakov, O., Chapman, J., Fahey, B., Gauthier, M.E.A., Mitros, T., *et al*. (2010) The *Amphimedon queenslandica* genome and the evolution of animal complexity. *Nature* **466**: 720–726.

Stamatakis, A. (2014) RAxML version 8: a tool for phylogenetic analysis and post-analysis of large phylogenies. *Bioinformatics* **30**: 1312–1313.

Tian, R.-M., Sun, J., Cai, L., Zhang, W.-P., Zhou, G.-W., Qiu, J.-W., and Qian, P.-Y. (2016) The deep-sea glass sponge *Lophophysema eversa* harbours potential symbionts responsible for the nutrient conversions of carbon, nitrogen and sulfur. *Environ Microbiol* **18**: 2481–2494.

Wang, H.-C., Susko, E., and Roger, A.J. (2006) On the correlation between genomic G+C content and optimal growth temperature in prokaryotes: Data quality and confounding factors. *Biochem Biophys Res Commun* **342**: 681–684.

Wiens, M., Korzhev, M., Perovic-Ottstadt, S., Luthringer, B., Brandt, D., Klein, S., and Muller, W.E.G. (2006) Toll-like receptors are part of the innate immune defense system of sponges (demospongiae: Porifera). *Mol Biol Evol* **24**: 792–804.

Wiens, M., Korzhev, M., Krasko, A., Thakur, N.L., Perović-Ottstadt, S., Breter, H.J., *et al*. (2005) Innate immune defense of the sponge *Suberites domuncula* against bacteria involves a MyD88-dependent signaling pathway: Induction of a perforin-like molecule. *J Biol Chem* **280**: 27949–27959.
